# Genetic Determinants of Trabecular and Cortical Volumetric Bone Mineral Densities and Bone Microstructure

**DOI:** 10.1371/journal.pgen.1003247

**Published:** 2013-02-21

**Authors:** Lavinia Paternoster, Mattias Lorentzon, Terho Lehtimäki, Joel Eriksson, Mika Kähönen, Olli Raitakari, Marika Laaksonen, Harri Sievänen, Jorma Viikari, Leo-Pekka Lyytikäinen, Dan Mellström, Magnus Karlsson, Östen Ljunggren, Elin Grundberg, John P. Kemp, Adrian Sayers, Maria Nethander, David M. Evans, Liesbeth Vandenput, Jon H. Tobias, Claes Ohlsson

**Affiliations:** 1 MRC Centre for Causal Analyses in Translational Epidemiology, University of Bristol, Bristol, United Kingdom; 2 School of Social and Community Medicine, University of Bristol, Bristol, United Kingdom; 3 Center for Bone and Arthritis Research, Institute of Medicine, Sahlgrenska Academy, University of Gothenburg, Gothenburg, Sweden; 4 Department of Clinical Chemistry, Fimlab Laboratories, University of Tampere and Tampere University Hospital, Tampere, Finland; 5 Department of Clinical Physiology, University of Tampere and Tampere University Hospital, Tampere, Finland; 6 Research Centre of Applied and Preventive Cardiovascular Medicine, University of Turku, Turku, Finland; 7 Department of Clinical Physiology and Nuclear Medicine, Turku University Hospital, Turku, Finland; 8 Department of Food and Environmental Sciences, University of Helsinki, Helsinki, Finland; 9 The UKK Institute for Health Promotion Research, Tampere, Finland; 10 Department of Medicine, University of Turku and Turku University Hospital, Turku, Finland; 11 Clinical and Molecular Osteoporosis Research Unit, Department of Orthopaedics, Lund University, Skane University Hospital, Malmö, Sweden; 12 Department of Medical Sciences, Uppsala University Hospital, Uppsala, Sweden; 13 Department of Human Genetics, McGill University, Montreal, Canada; 14 McGill University and Genome Quebec Innovation Centre, Montreal, Canada; 15 School of Clinical Sciences, University of Bristol, Bristol, United Kingdom; 16 Genomics Core Facility, University of Gothenburg, Gothenburg, Sweden; McGill University, Canada

## Abstract

Most previous genetic epidemiology studies within the field of osteoporosis have focused on the genetics of the complex trait areal bone mineral density (aBMD), not being able to differentiate genetic determinants of cortical volumetric BMD (vBMD), trabecular vBMD, and bone microstructural traits. The objective of this study was to separately identify genetic determinants of these bone traits as analysed by peripheral quantitative computed tomography (pQCT). Separate GWA meta-analyses for cortical and trabecular vBMDs were performed. The cortical vBMD GWA meta-analysis (*n = 5,878*) followed by replication (n = 1,052) identified genetic variants in four separate loci reaching genome-wide significance (*RANKL*, rs1021188, p = 3.6×10^−14^; *LOC285735*, rs271170, p = 2.7×10^−12^; *OPG*, rs7839059, p = 1.2×10^−10^; and *ESR1/C6orf97*, rs6909279, p = 1.1×10^−9^). The trabecular vBMD GWA meta-analysis (n = 2,500) followed by replication (n = 1,022) identified one locus reaching genome-wide significance (*FMN2/GREM2*, rs9287237, p = 1.9×10^−9^). High-resolution pQCT analyses, giving information about bone microstructure, were available in a subset of the *GOOD* cohort (n = 729). rs1021188 was significantly associated with cortical porosity while rs9287237 was significantly associated with trabecular bone fraction. The genetic variant in the *FMN2/GREM2* locus was associated with fracture risk in the MrOS Sweden cohort (HR per extra T allele 0.75, 95% confidence interval 0.60–0.93) and GREM2 expression in human osteoblasts. In conclusion, five genetic loci associated with trabecular or cortical vBMD were identified. Two of these (*FMN2/GREM2* and *LOC285735*) are novel bone-related loci, while the other three have previously been reported to be associated with aBMD. The genetic variants associated with cortical and trabecular bone parameters differed, underscoring the complexity of the genetics of bone parameters. We propose that a genetic variant in the *RANKL* locus influences cortical vBMD, at least partly, via effects on cortical porosity, and that a genetic variant in the *FMN2/GREM2* locus influences GREM2 expression in osteoblasts and thereby trabecular number and thickness as well as fracture risk.

## Author Summary

Osteoporosis is a common highly heritable skeletal disease characterized by reduced bone mineral density (BMD) and deteriorated bone microstructure, resulting in an increased risk of fracture. Most previous genetic epidemiology studies have focused on the genetics of the complex trait BMD, not being able to separate genetic determinants of the trabecular and cortical bone compartments and bone microstructure. The trabecular and cortical BMDs can be analysed separately by computed tomography. Therefore, we performed separate genome-wide association studies for trabecular and cortical BMDs, demonstrating that the genetic determinants of cortical and trabecular BMDs differ. Genetic variants in the *RANKL*, *LOC285735*, *OPG*, and *ESR1* loci were associated with cortical BMD, while a genetic variant in the *FMN2/GREM2* locus was associated with trabecular BMD. Two of these are novel bone-related loci. Follow-up analyses of bone microstructure demonstrated that a genetic variant in the *RANKL* locus is associated with cortical porosity and that the *FMN2/GREM2* locus is associated with trabecular number and thickness. We propose that a genetic variant in the *RANKL* locus influences cortical BMD via effects on cortical porosity, and that a genetic variant in the *FMN2/GREM2* locus influences trabecular BMD and fracture risk via effects on both trabecular number and thickness.

## Introduction

Meta-analyses of genome-wide association studies (GWAS) have identified a large number of loci associated with areal bone mineral density (aBMD) [1]–[6]. aBMD is a complex trait, obtained from a 2-dimensional projectional scan of the given bone with dual x-ray absorptiometry (DXA). Skeletal sites which are measured in this way, such as the lumbar spine and hip, comprise a mixture of cortical bone (compact bone comprising the outer shell), and trabecular bone (a network of thin interconnecting plates within the marrow cavity of vertebrae and the end of long bones). The lumbar spine has a relatively high proportion of trabecular bone, whereas the hip has a higher proportion of cortical bone. DXA-measured aBMD depends not only on bone cross-sectional size but also on apparent volumetric bone mineral density which is largely determined by trabecular microstructure and cortical thickness [7]. Although aBMD is the gold standard for diagnosing osteoporosis, it fails to provide a detailed skeletal phenotype necessary to discern traits such as trabecular volumetric BMD (vBMD), cortical vBMD and bone microstructural parameters. Previous studies using DXA have demonstrated that age is a major predictor of fracture risk independent of aBMD. Although this aBMD independent effect of age has been attributed to poor bone “quality”, the structural basis for this remains unclear [8]. Age-related changes in bone include microstructural deterioration, such as trabecular perforation, thinning, and loss of connectivity, as well as increased cortical porosity [8], [9]. Quantitative computed tomography (QCT) analysis has the capacity to reveal unique information about these bone traits.

Standard peripheral QCT (pQCT) with a resolution of 500 µm has the advantage of being able to separately analyse trabecular and cortical vBMDs. The correlation between trabecular and cortical vBMDs is low (r_s_ 0.11 in the young adult men of the GOOD cohort; [10]), supporting the notion that the determinants of these two bone parameters differ. Cortical vBMD but not trabecular vBMD reflects material density while trabecular vBMD mainly is influenced by trabecular number and thickness. In addition, the correlations of these vBMD parameters with femoral neck aBMD are low (cortical vBMD, r_s_ 0.04) or moderate (trabecular vBMD r_s_ 0.65), suggesting that cortical and trabecular vBMDs are at least partly influenced by genetic determinants not possible to identify by a GWAS of aBMD [10]. The heritability for trabecular vBMD has been reported to be as high as 59% while the heritability for cortical vBMD was slightly lower (40%) [11]. GWAS have revealed differences in genetic associations with lumbar and hip aBMD, providing some evidence that cortical and trabecular bone have distinct genetic influences [2].

We have in a previous smaller-scale GWAS meta-analysis (n = 1,934) identified a genetic variant in the *RANKL* locus to be significantly associated with cortical vBMD [10]. The genetic determinants of trabecular vBMD have not yet been evaluated using GWAS.

High resolution pQCT (HRpQCT) not only allows the separation of the trabecular and cortical bone compartments but also the assessment of bone microstructure. HRpQCT has an isotrophic voxel size of 82 µm and shows excellent correlation with *ex vivo* μCT imaging (resolution 20 µm or better) [8], [12], [13]. Importantly, HRpQCT analysis recently demonstrated that younger and older subjects with the same aBMD differed in cortical porosity, a key parameter not captured by DXA [8]. The genetic determinants of trabecular and cortical bone microstructure parameters as analysed by HRpQCT are unknown.

The objective of the present study was to identify genetic determinants of vBMDs and bone microstructure parameters separately for the cortical and trabecular bone compartments as analyzed by pQCT and HRpQCT. As our assembled discovery cohort was larger for the pQCT measurements (cortical vBMD n = 5,878, trabecular vBMD n = 2,500) than for the HRpQCT measurements (n = 729), we aimed to first identify genome-wide significant genetic variants for cortical and trabecular vBMDs separately and then to evaluate the impact of the identified variants on trabecular and cortical bone microstructure parameters in the HRpQCT cohort.

## Results

### Genome-wide association (GWA) meta-analyses of cortical and trabecular vBMDs

Table 1 displays the anthropometrics and bone traits for the four cohorts (ALSPAC discovery, GOOD baseline discovery, YFS discovery, and MrOS Sweden replication) evaluated. The association between cortical vBMD and trabecular vBMD was rather modest (Spearman's rank correlation coefficient [rho] *GOOD baseline* r = 0.11 [10]; *GOOD five year follow-up* r = −0.01). Separate GWA meta-analyses for cortical and trabecular vBMD were performed including all three discovery cohorts for cortical vBMD while trabecular vBMD was available in the YFS and GOOD cohorts.

**Table 1:** Characteristics of the cohorts included in the discovery GWA meta-analyses and replications.

	Discovery						Replication	
	ALSPAC		GOOD		YFS		MrOS Sweden	
	(n = 3382)		(n = 938)		(n = 1558)		(n = 1052)	
	mean	sd	mean	sd	mean	sd	mean	sd
Age, years	15.5	0.3	18.9	0.6	38	5.0	78.7	3.0
Men, %	47		100		44.5		100	
Height, cm	169.3	8.3	181.7	6.6	172.1	9.0	173.9	6.4
Weight, kg	61.2	11.3	73.9	11.6	77	16.4	79.2	11.2
*Cortical*								
Position of section	50%		25%		30%		38%	
vBMD, mg/cm^3^	1101	38	1156	20	1159	24	1128	40.8
*Trabecular*								
Position of section	NA	NA	4%		5%		4%	
vBMD, mg/cm^3^	NA	NA	266	34	241	34	217	37

Position = Position of section in proximal direction from distal end of tibia.

vBMD = volumetric bone mineral density; NA = not available.

### GWA meta-analysis of cortical vBMD

**Figure 1:**
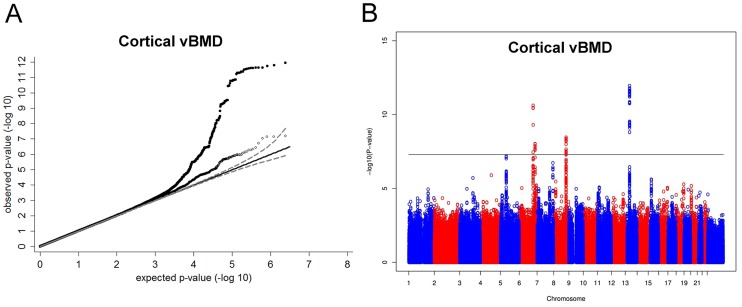
Genome-wide meta-analysis of cortical vBMD. (A) QQ plots of the genome-wide meta-analysis of cortical vBMD without (filled circles) or with (open diamonds) removal of the genome-wide significant loci (All SNPs excluded ±1 Mb around the hits). (B) Manhattan plot of the genome-wide meta-analysis of cortical vBMD.

**Figure 2:**
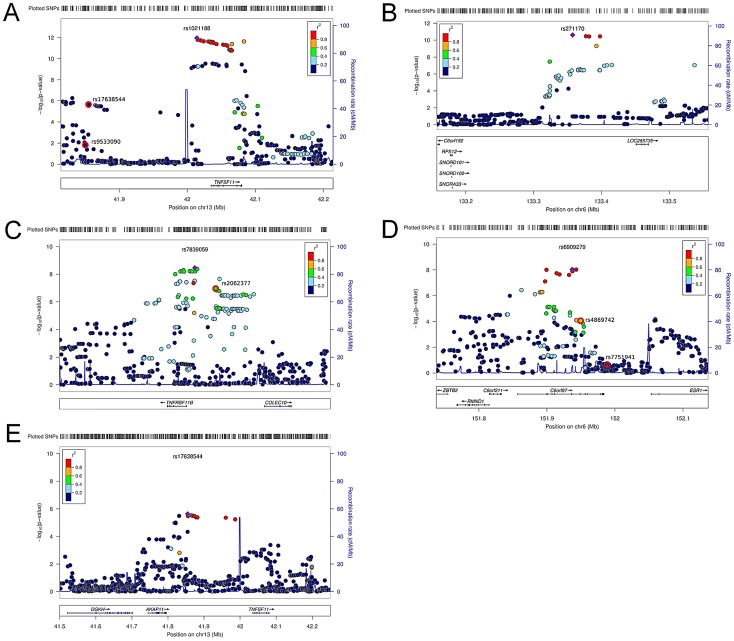
Regional association plots for the 5 independent signals from the discovery genome-wide meta-analysis of cortical vBMD. (A) rs1021188, (B) rs271170, (C) rs7839059, (D) rs6909279, (E) rs17638544. Circles show the GWA meta-analysis p-values, with different colors indicating varying linkage disequilibrium with the indicated SNP (diamond). SNPs in the same region identified in a recent large-scale GWA meta-analysis of aBMD are indicated by a red outer circle [2]. LocusZoom: http://csg.sph.umich.edu/locuszoom/.

Inverse variance weighted fixed-effect model meta-analysis of study-specific results was performed. In the cortical vBMD GWA meta-analysis of the ALSPAC, GOOD and YFS cohorts there was little systematic inflation of test statistics (Overall λ = 1.012 (1.023 for ALSPAC; 1.013 for GOOD; 1.023 for YFS)), but a marked deviation from the null distribution amongst the lowest observed p-values (Figure 1A). We identified genetic variants in four separate loci reaching genome-wide significance (Figure 1B). The greatest evidence for association between genetic variation and cortical vBMD was seen for rs1021188 (0.15 SD decrease per C allele; p = 1.4×10^−12^) on chromosome 13, slightly upstream of the *RANKL* gene (*TNFSF11*; Table 2, Figure 2A, Table S1). The second strongest genetic signal for cortical vBMD (rs271170; 0.11 SD decrease per T allele, p = 2.9×10^−11^) is a novel bone-related locus, located on chromosome 6, upstream of *LOC285735* (Table 2, Figure 2B, Table S1). The third strongest signal (rs7839059, 0.10 SD decrease per A allele, p = 4.1×10^−9^) was located on chromosome 8, upstream of *OPG* (*TNFRSF11B*; Table 2, Figure 2C, Table S1). The fourth genome-wide signal (rs6909279, 0.09 SD decrease per allele G, p = 1.0×10^−8^) was located on chromosome 6, in *C6orf97* upstream and close to *estrogen receptor-α* (*ESR1*; Table 2, Figure 2D, Table S1).

**Table 2:** Top cortical and trabecular vBMD signals from pQCT GWA meta-analyses followed by replication.

					Discovery				Replication					Combined			
					Meta-analysis				MrOS					All cohorts			
SNP	Closest gene	Chr	Effect allele	EAF	n	Beta	SE	P	n	EAF	Beta	SE	p	n	Effect	SE	p
*Cortical vBMD*																	
rs1021188	*TNFSF11*	13	C	0.17	5878	−0.15	0.02	1.4E-12	1052	0.15	−0.15	0.06	7.0E-03	6930	−0.15	0.02	3.6E-14
rs271170	*LOC285735*	6	T	0.33	5878	−0.11	0.02	2.9E-11	1025	0.29	−0.10	0.05	3.0E-02	6903	−0.11	0.02	2.7E-12
rs7839059	*TNFRSF11B*	8	A	0.34	5878	−0.10	0.02	4.1E-09	1025	0.33	−0.11	0.04	9.0E-03	6903	−0.10	0.02	1.2E-10
rs6909279	*C6orf97/ESR1*	6	G	0.40	5878	−0.09	0.02	1.0E-08	1027	0.38	−0.09	0.04	3.8E-02	6905	−0.09	0.01	1.1E-09
rs17638544*	*TNFSF11*	13	T	0.07	5873	0.13	0.03	4.2E-05	1009	0.06	0.18	0.09	3.8E-02	6882	0.13	0.03	5.1E-06
*Trabecular vBMD*																	
rs9287237	*FMN2*	1	T	0.15	2500	0.22	0.04	3.3E-08	1022	0.18	0.14	0.06	9.0E-03	3522	0.19	0.03	1.9E-09

Models adjusted for sex (ALSPAC and YFS), age, height, weight (ln). Betas in standard deviations and standard errors are presented.

* conditional adjusted for rs1021188; vBMD = volumetric bone mineral density; EAF = effect allele frequency.

We selected our top four regions and carried out analyses conditional on the most associated SNPs in each region. When conditioning on the most significant SNP in the *RANKL* region (rs1021188) an additional suggestive signal (rs17638544 close to *AKAP1* and upstream of *RANKL*, p = 4.2×10^−5^) appeared, but did not achieve genome-wide significance (Table 2, Figure 2E, Table S1). Using similar conditional analysis, no additional SNPs with an independent signal appeared in the other three evaluated regions (p<5×10^−5^).

The *RANKL*, *OPG* and *ESR1* regions have earlier been reported to be associated with aBMD in large scale GWA meta-analyses [1]–[6]. To evaluate if the identified SNPs associated with cortical vBMD in these regions are independent from the previously reported aBMD related SNPs, conditional analyses were performed (*RANKL region*, rs1021188 and rs17638544 were conditioned on the known aBMD hit rs9533090; *OPG region*, rs7839059 was conditioned on the known aBMD hit rs2062377; *ESR1 region*, rs6909279 was conditioned on known aBMD hits rs7751941 and rs4869742; [2]). The two cortical vBMD *RANKL* signals (rs1021188 and rs17638544) were distinct from the previously reported aBMD signal (rs9533090; [2]) in this region, supported by the fact that (i) rs9533090 was not significantly associated with cortical vBMD (Figure 2A), (ii) adjustment for rs9533090 did not influence the associations for rs1021188 or rs17638544 with cortical vBMD and the two cortical vBMD signals displayed a low r^2^ (<0.04) with rs9533090 (Table S2). It is difficult to determine if the identified cortical vBMD signal in the *OPG* region is separate from the previous reported aBMD signal in this region (rs2062377; [2]) as this previous aBMD signal also was significantly associated with cortical vBMD (Figure 2C), the r^2^ between the two SNPs was 0.39, and adjustment for rs2062377 slightly but not completely attenuated the association for rs7839059 with cortical vBMD (Table S2).

The identified cortical vBMD SNP in the *ESR1* region (rs6909279) is independent from one of the previous reported aBMD signals (rs7751941) while the other reported independent aBMD SNP in this region (rs4869742 [2]) displayed a relatively high r^2^ with rs6909279 (r^2^ = 0.60) (Figure 2D). However, adjustment for rs4869742 only slightly attenuated the association for rs6909279 with cortical vBMD (Table S2).

#### GWA meta-analysis of trabecular volumetric BMD

**Figure 3:**
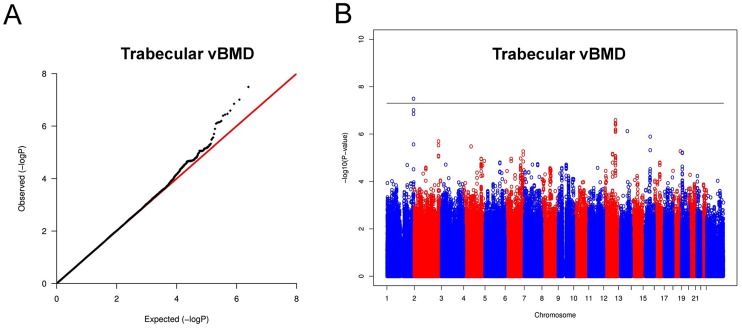
Genome-wide meta-analysis of trabecular vBMD. (A) QQ plot of the genome-wide meta-analysis of trabecular vBMD. (B) Manhattan plot of the genome-wide meta-analysis of trabecular vBMD.

**Figure 4:**
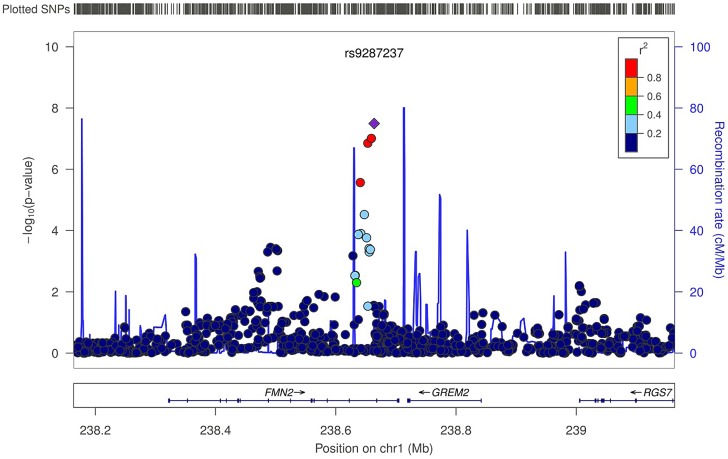
Regional association plot for rs9287237 of the discovery genome-wide meta-analysis of trabecular vBMD. Circles show the GWA meta-analysis p-values, with different colors indicating varying linkage disequilibrium with rs9287237 (diamond). LocusZoom: http://csg.sph.umich.edu/locuszoom/.

In the trabecular vBMD GWA meta-analysis there was little systematic inflation of test statistics (Overall λ = 1.005 (1.020 for GOOD; 1.018 for YFS)), but a significant deviation from the null distribution amongst the lowest observed p-values (Figure 3A). We identified one novel bone-related genetic variant reaching genome-wide significance (Figure 3B). The greatest evidence for association between genetic variation and trabecular vBMD was seen for rs9287237 (0.22 SD increase per T allele; p = 3.3×10^−8^) on chromosome 1, in the *formin 2* gene (*FMN2 gene*; Table 2, Figure 4, Table S1). When conditioning on the most significant SNP in the *FMN2 region*, no additional SNPs with an independent signal appeared (p<5×10^−5^).

The rs9287237 SNP significantly associated with trabecular vBMD was not associated with cortical vBMD, and three of the four genome-wide significant cortical vBMD SNPs were not significantly associated with trabecular vBMD (Table 3). However, the cortical vBMD SNP rs7839059 upstream of *OPG* displayed a modest association with trabecular vBMD (0.06 SD decrease per A allele; p = 4.7×10^−2^) in the same direction as seen for the genome-wide significant association with cortical vBMD (Table 3).

**Table 3:** Association of cortical and trabecular vBMD with top cortical and trabecular vBMD SNPs.

					Present pQCT meta-analysis								GEFOS aBMD meta-analysis							
					Cortical vBMD				Trabecular vBMD				FN BMD				LS BMD			
SNP	Closest gene	Chr	Effect allele	EAF	n	Beta	SE	P	n	Beta	SE	P	n^GEFOS^	EAF	Dir	P^GEFOS^	n^GEFOS^	EAF	Dir	P^GEFOS^
*Cortical vBMD SNPs*																				
rs1021188	*TNFSF11*	13	C	0.17	5952	−0.15	0.02	1.4E-12	2500	−0.01	0.04	8.5E-01	32961	0.14	−	1.7E-02	31800	0.14	−	2.2E-02
rs271170	*LOC285735*	6	T	0.33	5952	−0.11	0.02	2.9E-11	2500	0.02	0.03	5.4E-01	32961	0.29	+	5.4E-03	31800	0.29	+	6.9E-03
rs7839059	*TNFRSF11B*	8	A	0.34	5952	−0.10	0.02	4.1E-09	2500	−0.06	0.03	4.7E-02	32961	0.33	−	3.4E-17	31800	0.33	−	8.1E-16
rs6909279	*C6orf97/ESR1*	6	G	0.40	5952	−0.09	0.02	1.0E-08	2500	−0.04	0.03	2.0E-01	32961	0.46	−	2.5E-15	31800	0.46	−	5.3E-17
*Trabecular vBMD SNPs*																				
rs9287237	*FMN2*	1	T	0.15	5952	0.02	0.02	3.2E-01	2500	0.22	0.04	3.3E-08	32961	0.15	+	2.0E-04	31800	0.15	+	1.1E-03

Models adjusted for sex (ALSPAC and YFS), age, height, weight (ln). Betas in standard deviations and standard errors are presented. vBMD = volumetric bone mineral density; EAF = effect allele frequency; n^GEFOS^ and P^GEFOS^ is the number of subjects and p values as given from the GEFOS consortium (http://www.gefos.org/?q=content/data-release; [2]). Dir = Direction of the effect in the GEFOS publication using the same effect allele as in the present pQCT meta-analysis.

### Replication study of top SNPs for cortical and trabecular vBMD

#### Cortical vBMD

Five SNPs (four genome-wide significant [rs1021188, rs271170, rs7839059 and rs6909279] and one identified in the conditional analyses rs17638544) were selected for replication in the MrOS Sweden cohort. In this replication analysis all five SNPs were significantly associated with cortical vBMD in the same direction and with similar effect sizes as found in the discovery GWA meta-analysis (Table 2; Combined all cohorts, *rs1021188* 0.15 SD decrease per C allele, p = 3.6×10^−14^; *rs271170* 0.11 SD decrease per T allele, p = 2.7×10^−12^; *rs7839059* 0.10 SD decrease per A allele, p = 1.2×10^−10^; *rs6909279* 0.09 SD decrease per G allele, p = 1.1×10^−9^; rs17638544 0.13 SD increase per T allele, p = 5.1×10^−6^). The four GWA significant cortical vBMD SNPs rs1021188, rs271170, rs7839059 and rs6909279 explained 0.6%, 0.4%, 0.6% and 0.3%, respectively of the variation in cortical vBMD in the elderly men of the MrOS Sweden replication cohort. When including all four SNPs together, 1.5% of the variation in cortical vBMD was explained in the MrOS Sweden replication cohort. There was evidence for heterogeneity in the meta-analyses of the associations with rs1021188 (het p = 0.028) and rs17638544 (het p = 0.011). Random effects meta-analysis results for rs1021188 and rs17638544 were 0.18 SD per allele (SE = 0.05, 95% CI 0.08 to 0.28, p = 0.0003) and 0.14 SD per allele (SE = 0.07, 95% CI 0.00 to 0.28, p = 0.056), respectively.

#### Trabecular vBMD

One SNP (rs9287237) was replicated in the MrOS Sweden cohort and it was significantly associated with trabecular vBMD also in the replication cohort (Table 2; *Combined all cohorts*, 0.19 SD increase per T allele, p = 1.9×10^−9^). rs9287237 explained 0.7% of the variation in trabecular vBMD in the MrOS Sweden replication cohort.

### Sex-specific analyses

**Figure 5:**
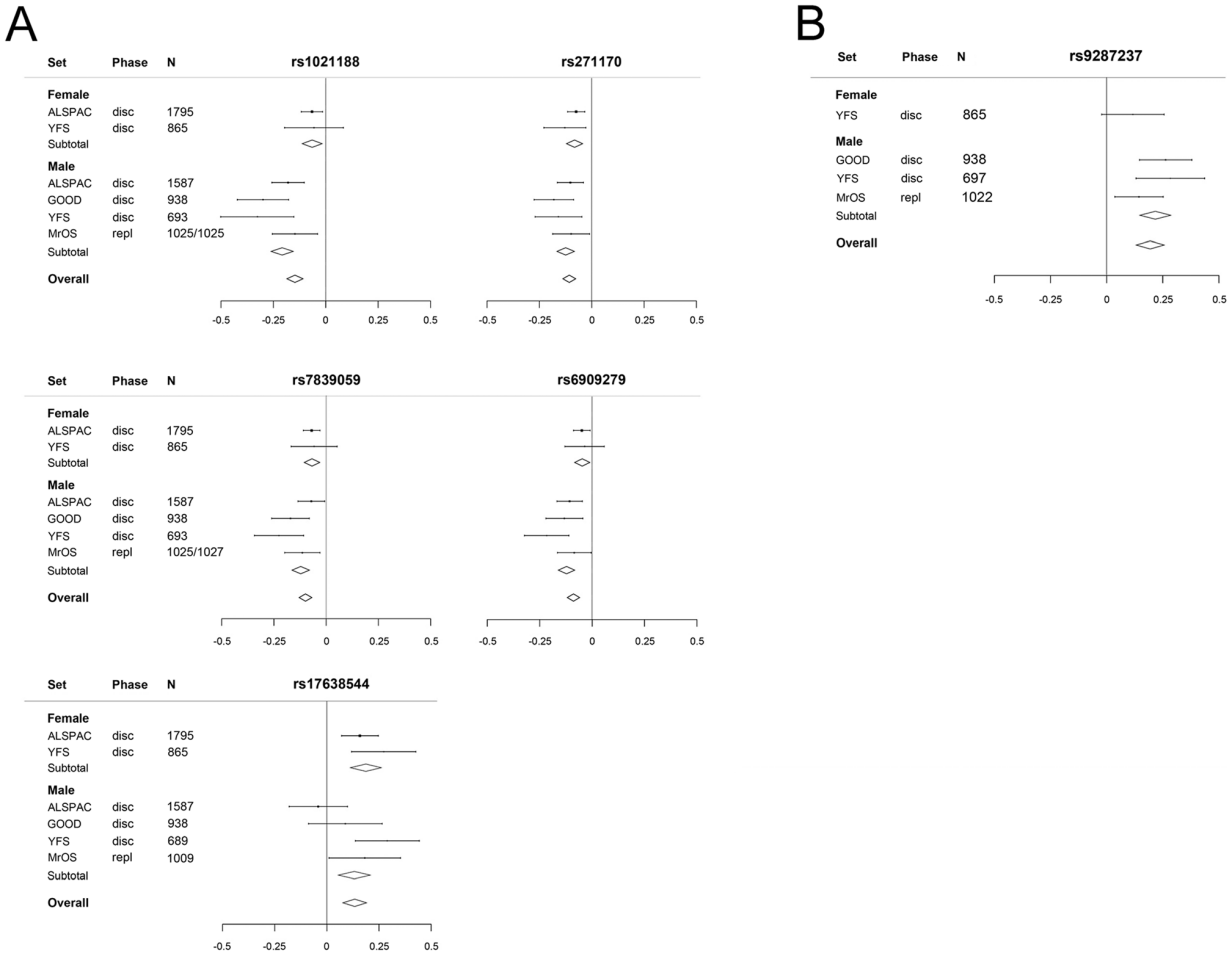
The genome-wide meta-analyses according to sex. (A) Cortical vBMD (*rs1021188* effect allele = C, *rs271170* effect allele = T, *rs7839059* effect allele = A, *rs6909279* effect allele = G, *rs17638544* effect allele = T). (B) Trabecular vBMD for *rs9287237* (effect allele = T). Mean and standard error vBMD z-scores are shown for each cohort, stratified by sex. Diamonds show the combined z-scores estimates per genotype (the width of the diamond represents the combined standard error). Disc = discovery cohort, repl = replication cohort.

Sex-specific analyses demonstrated that all five cortical vBMD SNPs were significantly associated with cortical vBMD in both men and women with effects in the same direction. However, the magnitude of the effect sizes differed significantly according to sex for three of the identified hits (Figure 5A; *rs1021188 in RANKL region*, men 0.21 SD and women 0.06 SD decrease per C allele, p = 4.3×10^−5^; *rs6909279 in ESR1 region*, men 0.12 SD and women 0.05 SD decrease per G allele, p = 3.0×10^−3^; *rs7839059 in OPG region*, men 0.12 SD and women 0.07 SD decrease per A allele, p = 3.0×10^−2^). The sex difference may explain the heterogeneity observed in the unstratified analyses for rs1021188 (both sexes have a het p>0.05). Significant heterogeneity is still observed in the males for rs17638544 (het p = 0.016).

The genetic variant associated with trabecular vBMD (rs9287237) was significantly associated with trabecular vBMD in men (Figure 5B, 0.22 SD increase per T allele) and a similar non-significant tendency in the same direction was observed in females (0.12 SD increase per T allele). It should be emphasized that for trabecular vBMD, we had a low statistical power to detect sex differences in effect sizes as the number of females included was rather low (n = 865).

### Genetic association versus HRpQCT measured bone microstructure

At the five year follow-up of the GOOD cohort both pQCT analysis, giving information about cortical and trabecular vBMD, and HRpQCT analyses, giving information about trabecular bone microstructure and cortical porosity, were available in the tibia for 729 subjects with genotype data available (Table 4). To determine the impact of the identified genome-wide significant cortical and trabecular vBMD signals for bone microstructure parameters, their associations with HRpQCT parameters were evaluated in the GOOD cohort. Trabecular vBMD as analysed by pQCT was strongly (r = 0.94) associated with trabecular bone fraction (BV/TV) as analysed by HRpQCT. The pQCT-derived cortical vBMD was moderately inversely correlated to cortical porosity as analysed by HRpQCT (r = −0.21).

**Table 4:** Characteristics of the GOOD five year follow-up cohort.

	mean	sd
Age, years	24.1	0.6
Men, no (%)	100	
Height, cm	182.4	6.5
Weight, kg	78.6	12.1
***pQCT*** * (n = 729)*		
Trabecular vBMD (mg/cm^3^)	261.7	35.5
Cortical vBMD (mg/cm^3^)	1163.3	19.3
***HRpQCT***		
*Trabecular (729)*		
BV/TV (%)	18.3	2.7
TbN (mm^−1^)	2.09	0.28
TbTh (µm)	88.1	11.1
TbSp (mm)	0.40	0.06
*Cortical (n = 725)*		
Porosity (%)	3.04	1.16

vBMD = volumetric bone mineral density; BV/TV = bone volume per total volume; TbN = trabecular number; TbTh = trabecular thickness; TbSp = trabecular separation.

#### Cortical vBMD SNPs

**Figure 6:**
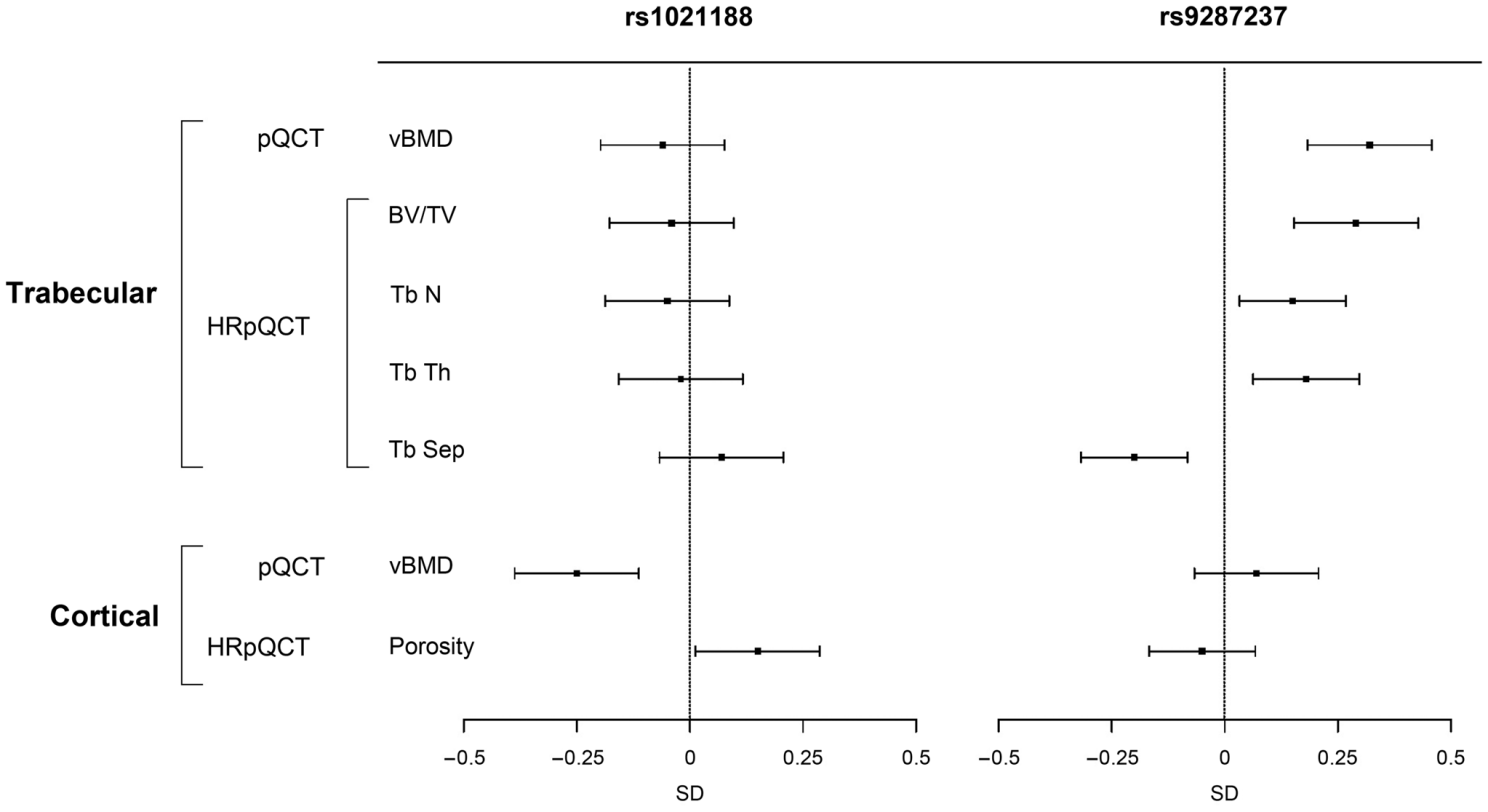
The associations of the SNPs explaining most of the cortical vBMD (rs1021188) and trabecular vBMD variations (rs9287237), respectively, with bone parameters in the GOOD cohort at the follow-up visit (n = 729). Mean and standard error z-scores are shown for trabecular and cortical vBMDs as analyzed by pQCT, and for trabecular bone volume per total volume (BV/TV), trabecular number (TbN), trabecular thickness (TbTh), trabecular separation (TbSp) and cortical porosity as analyzed by HRpQCT.

The four genome-wide significant cortical vBMD SNPs were all associated with (p<0.05) cortical but not trabecular vBMD at the five year follow-up visit of the GOOD cohort and their effect sizes for cortical vBMD were of similar magnitude and direction as seen for the GOOD cohort at the baseline visit (Tables S1 and S3, Figure 6). Interestingly, rs1021188, being the SNP explaining most of the cortical vBMD variation, was also associated with cortical porosity (0.15 SD increase per C allele, p = 3.0×10^−2^) but, as expected, in the inverse direction compared with the association with cortical vBMD (Figure 6 and Table S3).

#### Trabecular vBMD SNP

The genome-wide significant trabecular vBMD SNP rs9287237 was significantly associated with trabecular but not cortical vBMD at the five year follow-up visit of the GOOD cohort and the effect size (0.32 SD increase per T allele, p = 2.6×10^−6^) for trabecular vBMD was of similar magnitude and direction as seen for the GOOD cohort at the baseline visit (Tables S1 and S3, Figure 6). This SNP was also significantly associated with trabecular bone fraction (BV/TV) as analyzed by HRpQCT (0.29 SD increase per T allele, p = 1.8×10^−5^) while it was not significantly associated with cortical porosity (Figure 6). Detailed analysis of trabecular bone microstructure revealed that rs9287237 was not only associated with trabecular bone fraction but also with trabecular number (0.15 SD increase per T allele, p = 1.6×10^−2^), trabecular thickness (0.18 SD increase per T allele, p = 5.0×10^−3^) and trabecular spacing (0.20 SD decrease per T allele, p = 1.2×10^−3^; Figure 6).

### Estimation of the genetic correlation between cortical and trabecular vBMD

Although there appeared to be no overlap in the identity of the genome-wide significant SNPs between cortical and trabecular vBMD, it is still possible that there are genetic variants lower down the distribution of tests statistics which do not meet the stringent criteria for genome-wide significance, but nevertheless affect both traits pleiotropically. In order to investigate this possibility we ran a bivariate REML analysis using the GCTA software package in the GOOD cohort, having both cortical and trabecular vBMDs measurements available [14]. GCTA estimated the genetic correlation between trabecular and cortical BMD as rG = 0.0 (SE = 0.39) suggesting an absence of common genetic variants affecting both traits and consistent with our results from the genome-wide association analysis. However, we note that there are relatively few individuals in this analysis and consequently the standard errors on this estimate are very wide. In order to be more definitive with respect to the possible existence of pleiotropy one would need to perform the analysis in a much larger sample of individuals to yield precise estimates of the genetic correlation between the two traits.

### Comparison of the impact of identified genome-wide significant SNPs for vBMD and previously described aBMD SNPs

All five genome-wide significant vBMD SNPs were nominally significantly associated (p<0.05) with both femoral neck and lumbar spine aBMD as provided in the public data release from the discovery phase (n≅32,000) of the recent aBMD analyses from the GEFOS consortium (Table 3; http://www.gefos.org/?q=content/data-release) [2]. The direction of the effect was the same when comparing vBMDs and aBMD for four of the SNPs while it was opposite to the one described for aBMD for the cortical vBMD SNP rs271170.

When evaluating the 64 genome-wide significant aBMD SNPs recently identified by the GEFOS consortium [2] it was found that 15 of these were also significantly associated (p<0.05) with cortical vBMD and 15 were significantly associated with trabecular vBMD. Four of these SNPs were associated with both cortical and trabecular vBMDs (Table S4).

### eQTL analysis in human osteoblasts

In an attempt to assess the underlying functional mechanism of our identified loci we examined their potential role in regulating gene expression using expression quantitative trait locus (eQTL) data from resting (i.e. untreated) and induced (i.e. dexamethasone, BMP-2 and PGE2 treated) primary human osteoblasts [15], [16]. Expression of genes in close proximity to the five genome-wide significant SNPs (defined as located within the gene ±250 kb) was tested for association (Table S5). We found that the trabecular vBMD-associated SNP (rs9287237) was the strongest SNP significantly associated (P = 2.3×10^−4^) with expression of the nearby GREM2 gene. No significant effects on gene expression were noted at the additional four loci (Bonferroni adjusted P>0.05 corresponding to 0.05/88 = 5.7×10^−4^; Table S5).

### Association with fractures in MrOS Sweden

Overall, 388 men had at least one validated incident fracture after an average follow-up of 5.4 years in the MrOS Sweden cohort (Table S6). The trabecular vBMD SNP rs9287237, but none of the four cortical vBMD SNPs, was significantly associated with risk of all fractures (HR per extra T allele 0.75, 95% confidence interval (CI) 0.60–0.93) and hip fractures (HR per extra T allele 0.59, 95% CI 0.36–0.98; Table S7).

In two centers of MrOS Sweden, Gothenburg and Malmö, overall, 1445 men had a spine X-ray at baseline, which identified 225 men with prevalent vertebral fractures (15.6%; Table S6). The trabecular vBMD SNP rs9287237, but none of the four cortical vBMD SNPS, was significantly associated with prevalent X-ray verified vertebral fractures (OR per extra T allele 0.68, 95% CI 0.50–0.94; Table S7).

## Discussion

Osteoporosis is a common highly heritable skeletal disease characterized by reduced aBMD and deteriorated bone microstructure, resulting in an increased risk of fragility fracture [17]. Most previous genetic epidemiology studies have focused on the genetics of the complex trait aBMD, not being able to separate genetic determinants of trabecular vBMD, cortical vBMD and bone microstructure. However, all these structural traits are determinants of aBMD [7]. We, herein, provide evidence that the genetic determinants of cortical and trabecular vBMDs differ. Separate GWAS meta-analyses of cortical and trabecular vBMDs demonstrated that genetic variants in the *RANKL*, *LOC285735*, *OPG* and *ESR1* loci were associated with cortical vBMD while a genetic variant in the *FMN2/GREM2* locus was associated with trabecular vBMD. Follow-up analyses in a HRpQCT cohort providing bone microstructural traits revealed that a genetic variant in the *RANKL* locus is associated with not only cortical vBMD but also with cortical porosity. The *FMN2/GREM2* locus, on the other hand, is robustly associated with not only trabecular vBMD but also trabecular number and thickness as well as GREM2 expression in human osteoblasts and fracture risk.

Using the same pQCT cohorts as evaluated in the present study, we have recently identified a missense variant in the *WNT16* gene to be associated with cortical bone thickness [18]. Subsequent functional studies revealed that *Wnt16* inactivated mice had reduced cortical bone thickness, providing strong evidence that *WNT16* is an important regulator of cortical bone thickness. In the present discovery GWAS-meta-analysis, we identified genome-wide significant cortical vBMD signals in four loci and a trabecular vBMD signal in one locus and all these signals were significantly replicated in the MrOS Sweden cohort. One of the identified cortical vBMD loci (*LOC285735*) and the identified trabecular vBMD locus (*FMN2*) are novel bone-related loci while the remaining three identified cortical vBMD loci have been previously reported to be associated with aBMD. [2], [6], [19]. Although the number of subjects included in the present discovery GWAS meta-analyses of vBMD (cortical vBMD n = 5878; trabecular vBMD n = 2500) was substantially lower than the number of subjects included in a recent large-scale aBMD GWA meta-analysis (discovery cohort n = 32,961; [2]), the identification of novel bone-related loci in the trabecular and cortical vBMD analyses is not surprising as the correlations between aBMD on the one side and cortical and trabecular vBMDs on the other side are low/modest. In addition, the genetic variants associated with cortical and trabecular bone parameters, respectively, differed, and were also distinct from the *WNT16* locus recently identified to be associated with cortical bone thickness [18], underscoring the complexity of the genetics of bone parameters.

Conditional analysis revealed a secondary signal in the RANKL locus and both these cortical vBMD signals were independent of the previously reported aBMD signal (rs9533090; [2]) in this region, demonstrating that separate signals within the same region can have an impact on different bone traits ( = allelic heterogeneity). RANKL exerts its biological effects on bone by stimulating osteoclast differentiation following interactions with its receptor, RANK; how distinct genetic pathways might influence this functionality in different ways, so as to influence distinct phenotypic traits, is currently unclear. Alternatively, one of these signals may be in LD with a marker at a different gene responsible for mediating the genetic effect in question, or else represent a variant which although trans to a structural gene, affects transcription at other sites [20]. The cortical vBMD SNPs rs7839059 (*TNFRSF11B* locus) was also nominally (p<0.05) significantly associated with trabecular vBMD, although with less pronounced effect size, suggesting that this SNP does not exclusively have an impact on cortical bone. The present report describing two independent *RANKL* signals and one *OPG* signal with an impact on cortical vBMD provides further evidence that the RANK/RANKL/OPG axis affects the skeleton at least in part by influencing volumetric apparent density of cortical bone. It is tempting to speculate that changes in cortical vBMD contribute to the recent observations that the RANKL inhibitor denosumab reduces fracture risk [10], [21], [22]. Consistent with this possibility, administration of denosumab has been found to increase femoral cortical vBMD in mice with a knock-in of humanized RANKL [23].

The second strongest genetic signal for cortical vBMD was located on chromosome 6 (rs271170), 93.4 kb upstream of *LOC285735*. This is a novel bone-related signal and further targeted sequencing efforts and functional studies are required to characterize this signal. Several clinical and preclinical studies have clearly demonstrated that ESR1 is an important regulator of both female and male bone health [24]–[28] but the present study is first to provide genetic evidence that this receptor influences the volumetric apparent density of cortical bone. This finding is of importance as Khosla and co-workers recently proposed that the main physiological target for estrogen in bone is cortical and not trabecular bone [24].

A significant signal (rs9287237) for trabecular vBMD was identified on chromosome 1 located in the intron region of the *FMN2* gene. The combined effect size of this signal was substantial with an increase of 0.19 SD per T allele. *FMN2* is a gene that is expressed in oocytes and is required for progression through metaphase of meiosis 1 but it is not previously reported to influence the skeleton [29]. However, a genetic variant within *FMN2* has been associated with coronary heart disease [30]. The rs9287237 SNP is located slightly (55.7 kb) downstream of *GREM2 ( = PRDC)*, which is an extracellular antagonist of bone morphogenetic proteins (BMPs) and it inhibits osteoblastic differentiation [31], [32], making it an alternative plausible candidate gene underlying the rs9287237 association with trabecular vBMD. Importantly, eQTL analyses in human osteoblasts demonstrated that the trabecular vBMD-associated SNP (rs9287237) was significantly associated with expression of the nearby *GREM2* gene, indicating that GREM2 is a strong candidate for mediating the trabecular vBMD association at rs9287237. However, further targeted sequencing efforts and functional studies are required to characterize this signal.

There are known sex differences in bone traits in mice [33]–[36]. Similarly, some genome-wide linkage analyses in humans have reported sex-specific results. In a whole–genome linkage analysis stratified by sex, sex-specific QTLs were found in the Framingham sample [37]. Furthermore, in a meta-analysis that included data from nine whole-genome linkage scans for aBMD, several sex-specific QTLs were observed [38]. To our knowledge there is only one reported genome-wide significant aBMD signal, located on the X-chromosome (Xp22.31), which displays significant sex heterogeneity [2]. This signal was only significant in men and the same signal was also shown to be associated with male serum testosterone levels [39]. Sex-specific analyses in the present study revealed that all identified cortical vBMD signals were significantly associated with cortical vBMD in both men and women with effects in the same direction. Nevertheless, the magnitude of the effect sizes differed significantly according to sex for three of the identified hits. Importantly, the effect sizes of the *RANKL* and *ESR1* signals were more than three (0.21 SD vs. 0.06 SD) and two (0.12 vs. 0.05 SD) times larger, respectively, in men than in women. The smaller effect within females observed for rs1021188 in the *RANKL* region is mainly driven by ALSPAC, and there may be other reasons (such as younger age) why this study showed a smaller effect. However, the consistent results between ALSPAC and the YFS provide some evidence against the differences being driven primarily by age. The relative strong *ESR1* signal in men supports experimental and clinical studies, demonstrating that estrogens are crucial for male bone health [24], [25], [27], [40].

We examined genetic effects across cohorts encompassing a relatively broad age range, including 15 year old participants from ALSPAC who were still attaining peak bone mass, to older men from MrOS Sweden starting to show age-related bone loss. Inclusion of an older cohort had the advantage of providing an opportunity to study relationships with fracture risk. However, this design may have reduced the power to detect genetic associations by introducing greater heterogeneity. In contrast to aBMD, vBMD has been reported to change relatively little from adolescence to mid-life suggesting that analyses combining cohorts of different ages might be more informative when based on this trait [41]. However, recent follow up studies based on the GOOD cohort revealed substantial changes in cortical vBMD in the late teens and early twenties, at least in males [42]. Hence the suggestion that certain genetic associations with cortical vBMD were weaker in ALSPAC compared with other cohorts may reflect attenuation of effect during the consolidation of cortical bone whilst attaining peak bone mass.

Age-related changes in bone include microstructural deterioration, such as trabecular perforation, thinning, and loss of connectivity, as well as increased cortical porosity [8], [9]. These bone microstructural parameters are believed to have an aBMD-independent influence on fracture risk and they can be analyzed by HRpQCT. The present study is the first to identify genetic loci associated with cortical and trabecular bone microstructural parameters as analyzed by HRpQCT. The SNP in the *RANKL* region with the strongest association with cortical vBMD was also significantly associated with cortical porosity but, as expected, in the reverse direction. This finding suggests that a genetic variant in the *RANKL* locus influences cortical vBMD, at least partly, via effects on cortical porosity. Importantly, this signal in the RANKL region was independent from the previously reported aBMD signal in the same region [2]. Analyses of trabecular bone microstructure demonstrated that the trabecular vBMD SNP rs9287237 in the *FMN2/GREM2* locus was significantly associated with several trabecular but not cortical bone microstructure parameters. When evaluated in the five-year follow-up visit in the GOOD cohort, each T allele of this SNP resulted in a substantial increase in trabecular vBMD (0.32 SD), trabecular bone fraction (BV/TV 0.29 SD), trabecular number (0.15 SD), and trabecular thickness (0.18 SD). Thus, a genetic variant in the *FMN2/GREM2* locus influences trabecular vBMD via substantial effects on both trabecular number and thickness. Although, the present study is the first to report on genetic variants associated with microstructural bone-parameters, the analyses were candidate-based as a follow-up of our initial cortical and trabecular vBMD GWA meta-analyses. In order to identify novel genetic loci for bone microstructural parameters in a hypothesis-free manner, well-powered HRpQCT cohorts with genome-wide genotype data available need to be established.

We believe that our study provides strong evidence that previous large-scale GWA meta-analyses of the complex bone trait aBMD did not have the capability to identify a number of important loci with an impact on aspects of micro-architecture which may have important effects on fracture risk but be poorly reflected by overall aBMD measurements. We, therefore, propose that future well-powered pQCT and HRpQCT GWA meta-analyses of these specific bone structural traits will add useful information and might result in the identification of novel osteoporosis drug targets and provide novel aBMD-independent genetic markers for the prediction of fracture risk.

The implication of our results suggesting that cortical and trabecular bone compartments are under distinct genetic control is consistent with the fact that patients with idiopathic osteoporosis may present with a predominantly trabecular or cortical bone phenotype [43]. Although the lumbar spine and hip both comprise a mixture of bone types, the former has a relatively high proportion of trabecular bone, whereas the hip has a higher proportion of cortical bone. Hence, patients presenting with a disproportionate decrease in lumbar spine aBMD, which are well recognized, presumably have greater reductions in trabecular compared to cortical BMD [44]. Further studies are required to determine whether genetic variation in the *FMN2/GREM2* locus helps to explain this type of presentation.

The genetic variant in the *FMN2/GREM2* locus was associated with fracture risk and prevalent X-ray verified vertebral fractures in the MrOS Sweden cohort. However, further large-scale studies are required to replicate the fracture findings of this SNP. Collectively our data demonstrate that each extra T allele of rs9287237 is associated with decreased expression of the BMP antagonist GREM2 in osteoblasts, increased trabecular vBMD and decreased fracture risk. As previous *in vitro* studies have demonstrated that GREM2 inhibits osteoblast differentiation, we propose that rs9287237 is involved in the regulation of GREM2 expression in osteoblasts, which in turn regulates osteoblast differentiation and thereby the amount of trabecular bone and fracture risk [31], [32].

In conclusion, we identified five genetic loci associated with trabecular or cortical vBMD. Two of these (*FMN2/GREM2* and *LOC285735*) are novel bone-related loci while the other three have previously been reported to be associated with aBMD. The genetic variants reported to be associated with cortical and trabecular bone parameters differed and were also distinct from the *WNT16* locus recently identified to be associated with cortical thickness [18], underscoring the complexity of the genetics of bone parameters. Finally, we propose that a genetic variant in the *RANKL* locus influences cortical vBMD, at least partly, via effects on cortical porosity, and that a genetic variant in the *FMN2/GREM2* locus influences trabecular vBMD and fracture risk via substantial effects on both trabecular thickness and number.

## Materials and Methods

### Ethics statement

All study participants provided informed written consent. Approval by local institutional review boards was obtained in all studies.

### GOOD cohort

#### Participants

The Gothenburg Osteoporosis and Obesity Determinants (GOOD) study was initiated to determine both environmental and genetic factors involved in the regulation of bone and fat mass [45], [46]. Young, Caucasian men were randomly identified in the greater Gothenburg area in Sweden using national population registers, contacted by telephone, and invited to participate. Enrolled subjects were between 18 and 20 years of age. There were no other exclusion criteria, and 49% of the study candidates agreed to participate (n = 1,068). Five years later, the study subjects in the original GOOD study were contacted by letter and telephone and invited to participate in the 5-yr follow-up study. Of the original 1,068 subjects, 833 men, 24.1±0.6 yr of age, were included in the follow-up study (78% of the original population) [42]. The GOOD study was approved by the local ethics committee at University of Gothenburg. Oral and written informed consent was obtained from all study participants. Weight was measured to the nearest 0.1 kg and height was measured using a wall-mounted stadiometer.

#### pQCT measurements

Cortical volumetric BMD (not including the bone marrow) was measured on a single tibial diaphyseal slice (at 25% of the bone length in the proximal direction of the distal end) using the Stratec XCT2000 (Germany) at both the baseline visit and the five-year follow-up visit in the GOOD cohort [42], [46]. A threshold routine was used for defining cortical bone, which specified a voxel with a density >710 mg/cm^3^ as cortical bone. Trabecular vBMD (mg/cm^3^) was measured using a scan through the metaphysis (at 4% of the bone length in the proximal direction of the distal end) of the tibia. Tibia length was measured from the medial malleolus to the medial condyle. The CVs were <1% for all pQCT measurements. Both pQCT measurements and genotype information were available for 938 study subjects.

#### HRpQCT measurements

A high-resolution 3D pQCT device (XtremeCT; Scanco Medical AG, Brüttisellen, Switzerland) was used to scan the ultradistal tibia of the nondominant leg at the five-year follow-up visit in the GOOD cohort [47]. Anatomically formed carbon fiber shells, especially designed for each type of limb (Scanco Medical), were used to immobilize the subject's leg during the scan. The measurements of the volume of interest in the ultradistal tibia, 1 cm in the proximal direction and the whole cross-section in transversal direction, were carried out according to a standardized protocol previously described [47]. Briefly, a reference line was manually placed at the center of the end plate of the distal tibia. The first tomographic slice started 22.5 mm proximal to the reference line. A total of 110 parallel slices, with a nominal isotropic resolution (voxel size) of 82 µm, were obtained, delivering a 3D representation of an approximately 9-mm section of the tibia in the proximal direction. The entire volume of interest was automatically separated into cortical and trabecular region. From this separation and by previously described methods to process the data, we obtained trabecular bone volume fraction (BV/TV, percent), trabecular number (TbN; millimeters^−1^), trabecular thickness (TbTh; micrometers) and trabecular separation (TbSp; micrometers). For the cortical porosity, we used the manufacturer's software, which uses an approach published recently by several groups [48], [49] and described in detail by Burghardt and colleagues [8], [50]. The coefficients of variation (CVs) for the used bone measurements were obtained by three repeated measurements according to the standardized protocol on two subjects. The CVs ranged from 0.04 to 1.6%. Both HRpQCT measurements and genotype information were available for 729 study subjects.

#### Discovery set genotyping

Genotyping was performed with Illumina HumanHap610 arrays at the Genetic Laboratory, Department of Internal Medicine, Erasmus Medical Center, Rotterdam, the Netherlands. Genotypes were called using the BeadStudio calling algorithm. Genotypes from 938 individuals passed the sample quality control criteria (exclusion criteria: sample call rate <97.5%, excess autosomal heterozygosity >0.33 (FDR <0.1%), duplicates and/or first degree relatives identified using IBS probabilities (>97%), ethnic outliers (3 SD away from the population mean) using multi-dimensional scaling analysis with four principal coordinates). Across 22 duplicate samples, genotype concordance exceeded 99.9%. We carried out imputation to HapMap (NCBI build 36, release 21 and 22 for X chromosome and autosomes, respectively) (after excluding SNPs with MAF <1%, SNP call rate <98% and HWE p value<1×10^−6^) using Mach 1.0, Markov Chain Haplotyping, giving a total of 2,608,508 SNPs.

### YFS cohort

#### Participants

The Cardiovascular Risk in Young Finns Study (YFS) is an ongoing multi-centre follow-up of atherosclerosis risk factors in young Finns of Caucasian origin [51]. The first cross-sectional survey conducted in 1980 comprised a total of 3,596 subjects (83% of those invited) aged 3, 6, 9, 12, 15 and 18 years. The subjects were randomly selected from the national population register from five university cities in Finland (Helsinki, Turku, Tampere, Kuopio and Oulu) and the rural municipalities in their vicinity. In 2008, 1,884 subjects (1,058 women and 826 men) aged 31–46 years participated in pQCT measurements organized in five study centers (Turku, Helsinki, Tampere, Oulu and Kuopio) between February and December 2008. Trained technologists in each center performed the measurements. The same pQCT device was used in all study centers (Stratec XCT 2000R). Pregnant women were excluded from the pQCT measurements. Subjects gave written informed consent. The study protocol was approved by the local ethics committees of the participating universities and complied with national legislation.

#### pQCT measurements

Cortical volumetric BMD was measured on a single tibia diaphyseal slice (at 30% of the bone length in the proximal direction of the distal endplate ot the tibia) using the Stratec XCT2000 (Germany). A threshold routine was used for defining cortical bone, which specified a voxel with a density >710 mg/cm^3^ as cortical bone. Trabecular vBMD (mg/cm^3^) was measured using a scan through the metaphysis (at 5% of the bone length in the proximal direction of the distal endplate) of the tibia. Tibia length was measured from the medial malleolus to the medial condyle. The CVs were <2.6% for all pQCT measurements. Both pQCT measurements and genotype information were available for 1558 study subjects.

#### Discovery set genotyping

Genomic DNA was extracted from peripheral blood leukocytes using a commercially available kit and Qiagen BioRobot M48 Workstation according to the manufacturer's instructions (Qiagen, Hilden, Germany). Genotyping was done for 2556 samples using custom build Illumina Human 670 k BeadChip at the Welcome Trust Sanger Institute. Genotypes were called using the Illuminus clustering algorithm. 56 samples failed Sanger genotyping pipeline QC criteria (i.e., duplicated samples, heterozygosity, low call rate, or Sequenom fingerprint discrepancy). From the remaining 2500 samples one sample failed gender check, three were removed due to low genotyping call rate (<0.95) and 54 samples for possible relatedness (pi-hat>0.2). 11,766 SNPs were excluded based on HWE test (p< = 1×10^−6^), 7,746 SNPs failed a missingness test (call rate <0.95) and 34,596 SNPs failed frequency test (MAF<0.01). After quality control there were 2,442 samples and 546,677 genotyped SNPs available for further analysis. Genotype imputation was performed using MACH 1.0 and HapMap II CEU (NCBI build 36, release 21 and 22 for X chromosome and autosomes, respectively) samples as the reference set.

### ALSPAC cohort

#### Participants

The Avon Longitudinal Study of Parents and their Children (ALSPAC) is a geographically based birth cohort study investigating factors influencing the health, growth, and development of mainly Caucasian children [52]. All pregnant women resident within a defined part of the former county of Avon in South West England with an expected date of delivery between April 1991 and December 1992 were eligible for recruitment, of whom 14,541 were enrolled (http://www.alspac.bris.ac.uk). Both mothers and children have been extensively followed from the 8th gestational week onwards using a combination of self-reported questionnaires, medical records and physical examinations. Ethical approval was obtained from the ALSPAC Law and Ethics committee and relevant local ethics committees, written informed consent was provided by all parents. Blood samples were taken and DNA extracted as previously described [53]. Height was measured using a Harpenden stadiometer (Holtain Limited, Wales) and weight using a Tanita Body Fat Analyser.

#### pQCT measurements

pQCT scans were performed on approximately 4,500 children when they attended the age 15 research clinic. Cortical vBMD was measured on a single slice at the mid tibia (50%) using the Stratec XCT2000L (Germany). A threshold routine was used for defining cortical bone, which specified a voxel with a density >650 mg/cm^3^ as cortical bone. Of the 4500 pQCT scans obtained in ALSPAC 89 were rejected as being of insufficient quality. The CVs for cortical volumetric BMD based on 139 ALSPAC subjects scanned a mean of 31 days apart, was 1.3%.

#### Discovery set genotyping

9,912 ALSPAC individuals were genotyped using the Illumina HumanHap550 quad genome-wide SNP genotyping platform by 23andMe, subcontracting the Wellcome Trust Sanger Institute, Cambridge, UK and the Laboratory Corporation of America, Burlington, NC, US. Markers with <1% minor allele frequency >5% missing genotypes or which failed an exact test of Hardy-Weinberg equilibrium (HWE) (p<5×10^−7^) were excluded from further analysis. We also excluded any individuals who did not cluster with the CEU individuals in multidimensional scaling analysis, who had >3% missing data, minimal or excessive heterozygosity (>34.5% or <32% for the Sanger data and >33% or <31% for the LabCorp data), evidence of cryptic relatedness (>10% IBD) and any individuals with incorrect gender assignments. After data cleaning we were left with 8365 unrelated individuals with genome-wide genotyping, and 500,541 SNPs. We carried out imputation using MACH 1.0.16, Markov Chain Haplotyping, using CEPH individuals from phase 2 of the HapMap project as a reference set (NCBI build 36, release 21 and 22 for X chromosome and autosomes, respectively). Of the 8,365 individuals with imputed genotype data, 3,382 also had pQCT data.

### MrOS Sweden cohort (replication)

#### Participants

The Osteoporotic Fractures in Men (MrOS) study is a prospective multicenter study including older Caucasian men in Sweden (n = 3,014), Hong Kong (>2,000), and the United States (>6,000). In the present study, associations between candidate polymorphisms and skeletal parameters were investigated in the Swedish cohort, which consists of three sub-cohorts from three different Swedish cities (n = 1,005 in Malmö, n = 1,010 in Gothenburg, and n = 999 in Uppsala) [54]. Study subjects were randomly identified using national population registers, contacted and asked to participate. To be eligible for the study, the subjects had to be able to walk without assistance, provide self-reported data, and sign an informed consent; there were no other exclusion criteria. The study was approved by the ethics committees at the Universities of Gothenburg, Lund, and Uppsala. Informed consent was obtained from all study participants.

#### pQCT measurements

Cortical volumetric BMD (not including the bone marrow) was measured on a single tibial diaphyseal slice (at 38% of the bone length in the proximal direction of the distal end) using the Stratec XCT2000 (Germany) [10]. A threshold routine was used for defining cortical bone, which specified a voxel with a density >710 mg/cm^3^ as cortical bone. Trabecular vBMD (mg/cm3) was measured using a scan through the metaphysis (at 4% of the bone length in the proximal direction of the distal end) of the tibia. Tibia length was measured from the medial malleolus to the medial condyle. The CVs were <1% for all pQCT measurements. Adjustments for study centre were performed.

#### Replication set genotyping

Genotyping of SNPs identified in the GWAS meta-analysis was carried out at KBioscience using a competitive allele specific PCR (KASP) genotyping chemistry. The genotyping call rate was >97%.

### eQTL analysis in human osteoblasts

SNPs associated with vBMD at the genome-wide significance level as reported here were tested for association with resting or induced gene expression of neighbouring gene transcripts, in primary human osteoblasts derived from 113 (51 female and 62 male donors, respectively) unrelated Swedish donors. Detailed cell culture and analysis methods have been described in detail [15], [16]. Briefly, expression profiling of untreated, dexamethasone, BMP-2 and PGE2-treated cells each with up to three biological replicates was performed using the Illumina HumRef-8 BeadChips according to the protocol supplied by the manufacturer. Genotyping for genotype-expression association was performed using Illumina HapMap 550 k Duo chip. Individuals with low genotyping rate and SNPs showing significant deviation from Hardy-Weinberg equilibrium (P<0.05) were excluded. Similarly low frequency (MAF<0.05) SNPs and SNPs with high rates of missing data were excluded. Genotypes from samples that passed quality control (N = 103) were imputed for all SNPs (n = 478,805) oriented to the positive strand from phased (autosomal) chromosomes of the HapMap CEU Phase II panel (release 22, build 36) using MACH 1.0) [55]. A cut-off of R2<0.3 were used to remove poorly imputed markers. Association of imputed genotypes using estimated genotype probabilities with nearby expression traits (defined as ±250 kb window flanking the gene) were performed using a linear regression model implemented in the MACH2QTL software with sex and age as covariates.

### Assessment of incident fractures in MrOS Sweden

Participants were followed for 5.4 years on average after the baseline examination. The follow-up time was recorded from the date of the baseline visit to the date of the first fracture, the date of death, or end of the present follow-up interval. When a subject sustained a first fracture at different sites during the follow-up, the various fractures and the follow-up time for each respective first fracture type were included in the analyses. Complete follow-up was possible because central registers covering all Swedish citizens were used to identify the subjects and the time of death for all subjects who died during the study, and these analyses were performed after the time of fracture validation. At the time of fracture evaluation, the computerized X-ray archives in Malmö, Göteborg, and Uppsala were searched for new fractures occurring after the baseline visit, using the unique personal registration number, which all Swedish citizens have. All fractures reported by the study subject after the baseline visit were confirmed by physician review of radiology reports. Fractures reported by the study subject, but not possible to confirm by X-ray analyses report, were not included in this study. All validated fractures and hip fractures were evaluated. Information about the type of trauma associated with the incident fractures was not available. Fracture rates were expressed as the number of subjects with first fractures per 1000 person-years (Table S6).

### Assessment of prevalent vertebral fractures in MrOS Sweden

In Gothenburg and Malmö, 1445 men had an X-ray of the lateral thoracic and lumbar spine at baseline. All vertebral fractures were evaluated by an expert radiologist. If the vertebral body had a reduced height of 3 mm or more compared with the vertebra above, it was classified as a vertebral fracture [56].

### Statistical methods

The ALSPAC (n = 3382), YFS (n = 1558) and GOOD (n = 938) discovery cohorts contributed to the cortical vBMD genome-wide meta-analysis while the YFS and GOOD discovery cohorts contributed to the trabecular vBMD genome-wide meta-analysis. We analyzed only those imputed SNPs which had a minor allele frequency of >0.01 and an r^2^ imputation quality score of >0.3 in all 3 sets (n = 2,401,124). We carried out genome-wide association analyses for cortical and trabecular vBMDs using additive linear regression in Mach2QTL for ALSPAC, ProbABEL [57] for YFS and Mach2QTL on GRIMP [19] for the GOOD analyses. We included age, sex, height and weight(ln) as covariates. We carried out meta-analyses of the results from the three cohorts using the inverse variance method in METAL. Standardized betas and standard errors from each study were combined using a fixed effect model which weights the studies using the inverse variance and applying genomic control to individual studies and the combined results. Genome-wide significance was taken to be p<5×10^−8^. We also repeated the analyses in each of the three discovery cohorts, conditional on these top SNPs, to identify any additional independent associations in the regions. We selected one SNP for replication in the MrOS Sweden cohort from each independent region that had a p<5×10^−8^ as well as a secondary SNP from the RANKL region which appeared to influence cortical vBMD. Additive linear regression analyses were carried out for the associations between these SNPs and cortical and trabecular vBMDs in SPSS Statistics 17.0 for MrOS Sweden, using age, sex, height and weight(ln) as covariates. The results of all four cohorts were combined using a fixed effects inverse-variance meta-analysis in Stata (version 11.2). The SNPs showing evidence for heterogeneity (as assessed by a chi-squared test) were also meta-analysed using the DerSimonian & Laird random effects method. Correlations between bone traits in the GOOD cohort were tested and presented as Spearman's rank correlation coefficients (rho). The difference of the allelic association effects between males and females was tested using a two sample z-test.

Cox proportional hazards models were used to study the associations between SNPs and incident fractures. Prevalent vertebral fractures were analyzed using binary logistic regression models.

### Estimation of the genetic correlation between cortical and trabecular vBMD

In order to estimate the genetic correlation between cortical and trabecular vBMD we ran a bivariate REML analysis using the GCTA software package in the GOOD cohort, having both cortical and trabecular vBMDs measurements available [14]. This method essentially decomposes the covariance between the two traits into a part explained by common genetic variation that is tagged by markers on the SNP chip and a residual part that is not. It is then possible to use these estimates to estimate a genetic correlation between the two.

## Supporting Information

Table S1 Discovery meta-analysis of cortical and trabecular vBMDs.
(PDF)

Table S2 Cortical vBMD SNPs conditioned on known aBMD SNPs.
(PDF)

Table S3 Association of top cortical and trabecular vBMD signals with pQCT and HRpQCT parameters in the GOOD cohort at the five-year follow-up visit.
(PDF)

Table S4 Associations with cortical and trabecular vBMD for 64 reported genome-wide significant aBMD SNPs.
(PDF)

Table S5 eQTL analysis in human osteoblasts.
(PDF)

Table S6 Characteristics of the MrOS Sweden fracture cohort.
(PDF)

Table S7 Associations between cortical and trabecular vBMD SNPs and fractures in MrOS Sweden.
(PDF)
