# Supplementary material for: Genetic Determinants of Trabecular and Cortical Volumetric Bone Mineral Densities and Bone Microstructure
Source: PLoS Genet. 2013 Feb 21;9(2):e1003247. doi: 10.1371/journal.pgen.1003247 (PMC3578773; doi:10.1371/journal.pgen.1003247)
Supplement: Table S1 — Discovery meta-analysis of cortical and trabecular vBMDs. (PDF) [file pgen.1003247.s001.pdf]

Table S1. Discovery meta-analysis of cortical and trabecular vBMDs

					Alspac				GOOD				YFS				Meta-analysis			
**SNP**	**Closest gene**	**Chr**	**Effect allele**	**EAF**	**n**	**Beta**	**SE**	**P**	**n**	**Beta**	**SE**	**P**	**n**	**Beta**	**SE**	**P**	**n**	**Beta**	**SE**	**P**
Cortical vBMD																				
rs1021188	*TNFSF11*	13	C	0,17	3382	-0,12	0,02	2,0E-07	938	-0,30	0,06	1,7E-06	1558	-0,16	0,06	3,1E-03	5878	-0,15	0,02	1,4E-12
rs271170	*LOC285735*	6	T	0,33	3382	-0,09	0,02	3,1E-06	938	-0,18	0,05	1,8E-04	1558	-0,14	0,04	2,0E-04	5878	-0,11	0,02	2,9E-11
rs7839059	*TNFRSF11B*	8	A	0,34	3382	-0,07	0,02	1,0E-04	938	-0,17	0,05	2,1E-04	1558	-0,14	0,04	4,9E-04	5878	-0,10	0,02	4,1E-09
rs6909279	*C6orf97/ESR1*	6	G	0,40	3382	-0,07	0,02	6,2E-05	938	-0,13	0,05	3,2E-03	1558	-0,12	0,04	7,4E-04	5878	-0,09	0,02	1,0E-08
rs17638544*	*TNFSF11*	13	T	0,07	3382	0,06	0,04	1,5E-01	938	0,09	0,09	3,3E-01	1553	0,27	0,06	2,0E-06	5873	0,13	0,03	4,2E-05
Trabecular vBMD																				
rs9287237	*FMN2*	1	T	0,15	NA				938	0,26	0,06	1,0E-05	1562	0,19	0,05	3,9E-04	2500	0,22	0,04	3,3E-08

Models adjusted for sex (ALSPAC and YFS), age, height, weight (ln). Betas in standard deviations and standard errors are presented.
* conditional adjusted for rs1021188; vBMD = volumetric bone mineral density; NA = not available; EAF = effect allele frequency
