# Supplementary material for: Genetic Determinants of Trabecular and Cortical Volumetric Bone Mineral Densities and Bone Microstructure
Source: PLoS Genet. 2013 Feb 21;9(2):e1003247. doi: 10.1371/journal.pgen.1003247 (PMC3578773; doi:10.1371/journal.pgen.1003247)
Supplement: Table S2 — Cortical vBMD SNPs conditioned on known aBMD SNPs. (PDF) [file pgen.1003247.s002.pdf]

Table S2. Cortical vBMD SNPs conditioned on known aBMD SNPs

Region	SNP	Effect allele	n	Discovery cohort			Conditional Discovery cohort				
				Beta	SE	P	Conditioned on	Beta	SE	P	r^2
TNFSF11	rs1021188	C	5878	-0,147	0,021	1,4E-12	rs9533090	-0,151	0,020	8,0E-14	0,00
TNFSF11	rs17638544*	T	5873	0,129	0,031	4,2E-05	rs9533090	0,105	0,032	1,1E-03	0,04
TNFRSF11B	rs7839059	A	5878	-0,096	0,016	4,1E-09	rs2062377	-0,068	0,020	5,2E-04	0,39
C6orf97/ESR1	rs6909279	G	5878	-0,089	0,016	1,0E-08	rs7751941	-0,087	0,015	3,0E-09	0,00
							rs4869742	-0,086	0,022	6,3E-05	0,60

Models adjusted for sex (ALSPAC and YFS), age, height, weight (ln) with (conditional) or without further adjustment for previously known aBMD hits. Betas in standard deviations and standard errors are presented. r^2 = Linkage disequilibrium expressed as r^2 according to HapMap for cortical vBMD SNPs and earlier reported aBMD SNPs in the same region.
* conditional adjusted for rs1021188
