# Supplementary material for: Genetic Determinants of Trabecular and Cortical Volumetric Bone Mineral Densities and Bone Microstructure
Source: PLoS Genet. 2013 Feb 21;9(2):e1003247. doi: 10.1371/journal.pgen.1003247 (PMC3578773; doi:10.1371/journal.pgen.1003247)
Supplement: Table S3 — Association of top cortical and trabecular vBMD signals with pQCT and HRpQCT parameters in the GOOD cohort at the five-year follow-up visit. (PDF) [file pgen.1003247.s003.pdf]

Table S3. Asssociation of cortical and trabecular vBMD signals with pQCT and HRpQCT parameters in the GOOD cohort at the five-year follow-up visit

			pQCT								HRpQCT							
			Cortical vBMD				Trabecular vBMD				Cortical Porosity				BV/TV			
SNP	Chr	Effect allele	beta	SE	p	n	beta	SE	p	n	beta	SE	p	n	beta	SE	p	n
Cortical vBMD SNPs																		
rs1021188	13	C	**-0,25**	**0,07**	**6,0E-04**	**729**	-0,06	0,07	4,0E-01	728	**0,15**	**0,07**	**3,0E-02**	**725**	-0,04	0,07	5,5E-01	729
rs271170	6	T	**-0,20**	**0,05**	**2,1E-04**	**729**	-0,03	0,06	6,3E-01	728	0,04	0,05	5,0E-01	725	-0,05	0,06	3,8E-01	729
rs7839059	8	A	**-0,15**	**0,05**	**3,2E-03**	**729**	0,04	0,05	4,1E-01	728	0,03	0,05	4,9E-01	725	0,06	0,05	2,8E-01	729
rs6909279	6	G	**-0,14**	**0,05**	**4,8E-03**	**729**	-0,02	0,05	7,3E-01	728	-0,04	0,05	3,6E-01	725	0,03	0,05	5,7E-01	729
rs17638544	13	T	0,19	0,10	5,7E-02	729	-0,06	0,07	3,9E-01	728	-0,11	0,10	2,6E-01	725	-0,10	0,10	3,3E-01	729
Trabecular vBMD SNPs																		
rs9287237	1	T	0,07	0,07	3,3E-01	729	**0,32**	**0,07**	**2,6E-06**	**728**	-0,05	0,06	4,5E-01	725	**0,29**	**0,07**	**1,8E-05**	**729**

Models adjusted for age, height, weight(ln) Betas in standard deviations and standard errors are presented.
vBMD = volumetric bone mineral density; bold = p< 0.05
