# Supplementary material for: Genetic Determinants of Trabecular and Cortical Volumetric Bone Mineral Densities and Bone Microstructure
Source: PLoS Genet. 2013 Feb 21;9(2):e1003247. doi: 10.1371/journal.pgen.1003247 (PMC3578773; doi:10.1371/journal.pgen.1003247)
Supplement: Table S4 — Associations with cortical and trabecular vBMD for 64 reported genome-wide significant aBMD SNPs. (PDF) [file pgen.1003247.s004.pdf]

Table S4 Associations with cortical and trabecular vBMD for 64 reported genome-wide significant aBMD SNPs [2]

SNP	Locus	Closest Gene	EA	GEFOS aBMD meta-analyses					Cortical vBMD				Trabecular vBMD			
				Dir	FN aBMD		LS aBMD									
					$Effect^{GEFOS}$	P^{GEFOS}	$Effect^{GEFOS}$	P^{GEFOS}	EAF	Effect	SE	P	EAF	Effect	SE	P
rs12407028	1p31.3	WLS	t	+	0,05	3,4E-23	0,08	3,1E-45	0,59	0,01	0,02	6,0E-01	**0,57**	**0,07**	**0,03**	**1,4E-02**
rs17482952	1p31.3	WLS	a	+	0,08	1,3E-11	0,07	1,7E-08	0,92	0,04	0,03	1,6E-01	0,92	0,03	0,05	5,1E-01
rs7521902	1p36.12	WNT4	a	-	-0,04	2,8E-09	-0,05	9,7E-11	**0,24**	**-0,06**	**0,02**	**1,7E-03**	0,25	-0,01	0,03	7,2E-01
rs6426749	1p36.12	ZBTB40	c	+	0,11	7,4E-57	0,10	1,9E-44	0,17	0,01	0,02	7,7E-01	**0,12**	**0,11**	**0,04**	**1,5E-02**
rs479336	1q24.3	DNM3	t	-	-0,04	8,5E-15	-0,03	2,1E-05	0,75	0,00	0,02	8,1E-01	0,74	-0,04	0,03	2,5E-01
rs4233949	2p16.2	SPTBN1	c	+	0,02	5,9E-06	0,05	2,2E-18	**0,38**	**0,03**	**0,02**	**4,5E-02**	0,32	0,02	0,03	6,1E-01
rs7584262	2p21	PKDCC	t	+	0,04	1,3E-09	0,01	7,0E-02	0,23	-0,01	0,02	6,4E-01	**0,24**	**0,07**	**0,03**	**4,5E-02**
rs17040773	2q13	ANAPC1	a	+	0,04	1,5E-09	0,01	1,9E-01	0,77	-0,03	0,02	1,8E-01	0,80	0,02	0,04	5,7E-01
rs1878526	2q14.2	INSIG2	a	+	0,00	7,9E-01	0,04	1,2E-10	0,20	0,01	0,02	6,3E-01	0,16	0,02	0,04	6,1E-01
rs1346004	2q24.3	GALNT3	a	-	-0,05	1,1E-25	-0,06	3,9E-30	**0,51**	**-0,04**	**0,02**	**2,3E-02**	**0,53**	**-0,06**	**0,03**	**5,0E-02**
rs430727	3p22.1	CTNNB1	t	-	-0,06	4,4E-25	-0,05	1,5E-18	0,45	0,03	0,02	6,1E-02	0,44	-0,06	0,03	5,4E-02
rs1026364	3q13.2	KIAA2018	t	+	0,03	4,1E-10	0,02	7,6E-04	0,36	0,00	0,02	8,7E-01	**0,41**	**0,08**	**0,03**	**5,9E-03**
rs344081	3q25.31	LEKR1	t	+	0,04	2,2E-06	0,06	4,5E-12	0,86	0,00	0,02	8,7E-01	0,84	-0,01	0,04	7,9E-01
rs3755955	4p16.3	IDUA	a	-	-0,06	1,5E-14	-0,06	5,2E-15	0,15	-0,04	0,02	5,2E-02	0,16	-0,05	0,04	1,9E-01
rs6532023	4q22.1	MEPE	t	+	0,06	4,9E-26	0,06	1,2E-27	0,33	0,01	0,02	5,6E-01	0,31	0,02	0,03	6,0E-01
rs1366594	5q14.3	MEF2C	a	+	0,08	4,5E-61	0,01	1,0E-02	0,55	-0,03	0,02	5,8E-02	0,57	0,04	0,03	1,6E-01
rs11755164	6p21.1	SUPT3H	t	-	-0,01	5,0E-02	-0,04	5,6E-11	**0,42**	**-0,05**	**0,02**	**2,7E-03**	0,41	-0,03	0,03	3,2E-01
rs9466056	6p22.3	CDKAL1	a	-	-0,04	2,7E-13	-0,03	3,6E-08	0,38	-0,01	0,02	4,8E-01	0,36	0,03	0,03	3,8E-01
rs13204965	6q22.32	RSPO3	a	+	0,04	8,1E-12	0,04	3,6E-10	0,77	0,01	0,02	6,1E-01	0,79	-0,02	0,04	5,2E-01
rs7751941	6q25.1	C6orf97	a	-	-0,04	1,6E-09	-0,08	2,0E-24	0,21	-0,02	0,02	2,2E-01	0,18	-0,05	0,04	1,8E-01
rs4869742	6q25.1	C6orf97	t	-	-0,05	4,1E-18	-0,08	3,9E-35	**0,29**	**-0,07**	**0,02**	**9,6E-05**	0,29	-0,06	0,03	5,4E-02
rs10226308	7p14.1	TXNDC3	a	-	-0,02	2,0E-02	-0,06	6,4E-13	0,82	-0,02	0,02	3,4E-01	0,82	-0,02	0,04	6,0E-01
rs6959212	7p14.1	STARD3NL	t	-	-0,04	1,2E-13	-0,07	3,8E-38	0,32	-0,02	0,02	1,5E-01	0,29	-0,04	0,03	1,8E-01
rs4727338	7q21.3	SLC25A13	c	+	0,08	8,1E-48	0,07	2,1E-35	**0,67**	**0,06**	**0,02**	**9,7E-04**	0,70	0,05	0,03	1,3E-01
rs3801387	7q31.31	WNT16	a	-	-0,08	5,0E-40	-0,09	3,2E-51	**0,74**	**-0,04**	**0,02**	**1,6E-02**	**0,76**	**-0,08**	**0,03**	**1,0E-02**
rs13245690	7q31.31	C7orf58	a	+	0,02	8,2E-04	0,05	1,6E-11	**0,63**	**0,07**	**0,02**	**2,4E-05**	***0,68***	***-0,07***	***0,03***	***1,4E-02***
rs7812088	7q36.1	ABCF2	a	+	0,05	7,3E-09	0,04	2,2E-07	0,13	0,03	0,02	1,4E-01	0,15	0,07	0,04	8,8E-02
rs7017914	8q13.3	XKR9	a	+	0,03	2,3E-07	-0,01	2,6E-01	0,49	-0,02	0,02	1,4E-01	0,48	0,04	0,03	1,2E-01
rs2062377	8q24.12	TNFRSF11B	a	-	-0,06	9,1E-25	-0,08	3,2E-39	**0,57**	**-0,08**	**0,02**	**1,4E-07**	0,57	-0,04	0,03	1,3E-01
rs7851693	9q34.11	FUBP3	c	+	0,05	3,4E-22	0,03	6,1E-08	0,63	-0,01	0,02	5,0E-01	0,60	0,05	0,03	9,1E-02
rs3905706	10p11.23	MPP7	t	+	-0,01	3,0E-02	0,05	2,4E-16	0,21	-0,01	0,02	4,8E-01	0,20	0,02	0,03	5,9E-01
rs1373004	10q21.1	MBL2	t	-	-0,04	1,4E-08	-0,06	1,6E-12	0,14	-0,05	0,02	5,3E-02	**0,15**	**-0,14**	**0,04**	**8,4E-04**
rs7071206	10q22.3_1	KCNMA1	t	-	0,00	8,1E-01	-0,06	5,0E-19	**0,80**	**-0,04**	**0,02**	**4,2E-02**	0,81	0,02	0,04	5,6E-01
rs7084921	10q24.2	CPN1	t	+	0,03	9,0E-10	0,03	9,1E-07	0,39	0,02	0,02	2,2E-01	***0,32***	***-0,07***	***0,03***	***2,5E-02***
rs7932354	11p11.2	ARHGAP1	t	+	0,05	5,1E-18	0,04	5,4E-12	0,31	0,01	0,02	4,3E-01	0,33	0,05	0,03	6,4E-02
rs10835187	11p14.1_1	LIN7C	t	-	-0,01	3,0E-02	-0,03	4,9E-08	0,55	-0,01	0,02	6,4E-01	0,54	-0,01	0,03	8,3E-01
rs163879	11p14.1_2	DCDC5	t	-	-0,03	2,1E-08	-0,04	2,2E-11	0,66	0,00	0,02	8,7E-01	**0,61**	**-0,06**	**0,03**	**4,7E-02**
rs7108738	11p15.2	SOX6	t	-	-0,08	1,1E-32	-0,03	2,1E-06	**0,79**	**-0,05**	**0,02**	**1,7E-02**	**0,73**	**-0,08**	**0,03**	**2,0E-02**
rs3736228	11q13.2	LRP5	t	-	-0,05	4,8E-11	-0,08	2,1E-26	0,14	-0,03	0,02	1,3E-01	0,09	-0,09	0,05	1,0E-01
rs7953528	12p11.22	KLHDC5	a	+	0,05	1,9E-12	-0,01	1,3E-01	0,17	-0,01	0,02	5,4E-01	0,16	0,05	0,04	2,2E-01
rs2887571	12p13.33	ERC1	a	-	-0,03	6,5E-09	-0,04	5,6E-12	0,76	0,00	0,02	9,2E-01	0,79	-0,02	0,03	5,7E-01
rs12821008	12q13.12	DHH	t	+	0,03	3,3E-07	0,05	1,2E-15	0,42	0,01	0,02	3,8E-01	**0,44**	**0,06**	**0,03**	**4,5E-02**
rs736825	12q13.13	HOXC6	c	+	0,04	1,1E-09	0,05	7,7E-16	***0,61***	***-0,04***	***0,02***	***1,6E-02***	0,59	0,04	0,03	1,8E-01
rs2016266	12q13.13	SP7	a	-	-0,03	3,7E-10	-0,05	2,9E-20	0,68	-0,01	0,02	4,3E-01	0,69	-0,01	0,03	7,0E-01
rs1053051	12q23.3	C12orf23	t	-	-0,03	9,6E-10	-0,03	7,9E-08	0,51	0,01	0,02	4,6E-01	0,50	0,03	0,03	2,5E-01
rs9533090b	13q14.11	AKAP11	t	-	-0,05	4,9E-23	-0,10	4,8E-68	**0,50**	**-0,04**	**0,02**	**2,0E-02**	0,52	-0,04	0,03	1,2E-01
rs1286083	14q32.12	RPS6KA5	t	-	-0,05	2,0E-15	-0,05	1,7E-14	0,82	0,01	0,02	8,0E-01	0,83	0,00	0,04	9,8E-01
rs11623869	14q32.32	MARK3	t	-	-0,04	5,2E-16	-0,04	5,1E-11	***0,35***	***0,04***	***0,02***	***1,0E-02***	0,33	-0,03	0,03	3,4E-01
rs4985155	16p13.11	NTAN1	a	-	-0,03	1,7E-10	-0,03	2,1E-09	0,66	-0,01	0,02	7,2E-01	0,66	-0,04	0,03	1,6E-01
rs9921222	16p13.3_1	AXIN1	t	-	-0,04	5,2E-12	-0,04	1,0E-16	0,48	0,01	0,02	3,4E-01	0,47	-0,04	0,03	1,4E-01
rs13336428	16p13.3_2	C16orf38	a	-	-0,04	1,5E-16	-0,04	1,7E-13	0,41	-0,02	0,02	2,0E-01	0,40	-0,05	0,03	6,6E-02
rs1566045	16q12.1	SALL1	t	-	-0,06	1,9E-22	-0,01	4,0E-02	0,80	0,01	0,02	6,9E-01	**0,84**	**-0,14**	**0,04**	**3,0E-04**
rs1564981	16q12.1	CYLD	a	-	-0,02	4,4E-05	-0,04	1,9E-10	0,45	0,01	0,02	4,5E-01	0,43	-0,02	0,03	4,2E-01
rs10048146	16q24.1	FOXL1	a	+	0,05	1,0E-14	0,05	3,1E-11	0,81	0,02	0,02	2,9E-01	0,80	0,00	0,04	9,9E-01
rs4790881	17p13.3	SMG6	a	+	0,05	9,7E-19	0,03	3,4E-09	0,69	0,02	0,02	2,8E-01	0,69	0,05	0,03	1,1E-01
rs4792909	17q21.31_1	SOST	t	+	0,04	1,9E-11	0,04	9,4E-10	0,41	0,00	0,02	8,3E-01	**0,46**	**0,07**	**0,03**	**1,3E-02**
rs227584	17q21.31_1	C17orf53	a	-	-0,06	2,6E-24	-0,04	9,9E-10	0,70	0,03	0,02	6,6E-02	0,68	0,00	0,03	9,0E-01
rs1864325	17q21.31_2	MAPT	t	-	-0,03	7,5E-05	-0,04	4,9E-11	0,22	0,01	0,02	5,3E-01	**0,13**	**-0,11**	**0,05**	**1,2E-02**
rs7217932	17q24.3	SOX9	a	+	0,03	1,9E-11	0,01	8,0E-02	0,48	0,01	0,02	7,0E-01	0,49	0,04	0,03	1,8E-01
rs4796995	18p11.21	C18orf19	a	+	0,03	4,8E-08	0,02	6,6E-04	0,63	0,01	0,02	4,4E-01	0,62	0,02	0,03	6,0E-01
rs884205	18q21.33	TNFRSF11A	a	-	-0,04	3,2E-10	-0,05	1,6E-17	**0,29**	**-0,06**	**0,02**	**6,0E-04**	0,30	-0,04	0,03	2,2E-01
rs10416218	19q13.11	GPATCH1	t	-	-0,03	5,5E-08	-0,04	6,6E-11	0,75	0,01	0,02	4,3E-01	0,75	-0,02	0,03	6,2E-01
rs3790160	20p12.2	JAG1	t	+	0,04	3,6E-12	0,05	3,1E-19	0,50	0,01	0,02	4,2E-01	0,47	0,03	0,03	2,4E-01
rs5934507	Xp22.31	FAM9B	a	-	-0,01	3,0E-02	-0,02	7,3E-03	0,66	0,05	0,02	3,3E-04	0,54	0,02	0,03	4,2E-01

Models adjusted for sex (ALSPAC and YFS), age, height, weight (ln). Betas in standard deviations and standard errors are presented. vBMD = volumetric bone mineral density; EA = effect allele; EAF = effect allele frequency; Dir = Direction of the effect in GEFOS publication using the same effect allele as in the present pQCT meta-analysis [2]. $Effect^{GEFOS}$ and P^{GEFOS} are the effect sizes and p values as given from the GEFOS consortium [2], Bold = p<0.05. Italics = opposite direction of effect vs that seen for aBMD
