# Supplementary material for: Genetic Determinants of Trabecular and Cortical Volumetric Bone Mineral Densities and Bone Microstructure
Source: PLoS Genet. 2013 Feb 21;9(2):e1003247. doi: 10.1371/journal.pgen.1003247 (PMC3578773; doi:10.1371/journal.pgen.1003247)
Supplement: Table S5 — eQTL analysis in human osteoblasts. (PDF) [file pgen.1003247.s005.pdf]

Table S5 eQTL analysis in human osteoblasts

ProbeID	GeneID	SNPID	alleles (A,B)	freq (A)	beta(A)	SE	p-value	treatment
ILMN_24616	GREM2	rs9287237	T,G	0,16	-0,14	0,04	2,3E-04	dex
ILMN_941	C6orf211	rs6909279	C,G	0,60	-0,06	0,02	3,9E-03	untreated
ILMN_24099	EYA4	rs271170	C,T	0,70	-0,18	0,07	1,3E-02	bmp2
ILMN_6397	COLEC10	rs7839059	A,C	0,32	0,01	0,00	2,0E-02	pge2
ILMN_24616	GREM2	rs9287237	T,G	0,84	-0,12	0,06	4,4E-02	pge2
ILMN_18367	FMN2	rs9287237	G,T	0,84	0,22	0,12	5,7E-02	dex
ILMN_18218	C6orf97	rs6909279	C,G	0,60	0,02	0,01	6,0E-02	bmp2
ILMN_9320	AKAP12	rs6909279	C,G	0,60	-0,12	0,07	7,1E-02	untreated
ILMN_23727	MAL2	rs7839059	A,C	0,32	0,01	0,01	7,7E-02	dex
ILMN_6233	C6orf192	rs271170	C,T	0,70	0,07	0,04	7,8E-02	bmp2
ILMN_18218	C6orf97	rs6909279	C,G	0,60	0,02	0,01	8,2E-02	dex
ILMN_21404	NOV	rs7839059	A,C	0,32	-0,21	0,12	8,5E-02	dex
ILMN_6233	C6orf192	rs271170	C,T	0,70	0,09	0,05	9,0E-02	pge2
ILMN_6233	C6orf192	rs271170	C,T	0,70	0,07	0,04	9,1E-02	untreated
ILMN_14698	FLJ40919	rs1021188	C,T	0,15	-0,01	0,01	9,5E-02	untreated
ILMN_24099	EYA4	rs271170	C,T	0,70	-0,12	0,07	9,8E-02	untreated
ILMN_9320	AKAP12	rs6909279	C,G	0,60	-0,09	0,06	9,9E-02	pge2
ILMN_18367	FMN2	rs9287237	G,T	0,84	0,17	0,11	1,1E-01	pge2
ILMN_16767	DGKH	rs1021188	C,T	0,15	0,01	0,00	1,1E-01	pge2
ILMN_24099	EYA4	rs271170	C,T	0,70	-0,13	0,08	1,2E-01	dex
ILMN_6233	C6orf192	rs271170	C,T	0,70	0,08	0,05	1,2E-01	dex
ILMN_23727	MAL2	rs7839059	A,C	0,32	0,02	0,01	1,3E-01	untreated
ILMN_14698	FLJ40919	rs1021188	C,T	0,15	-0,01	0,01	1,3E-01	bmp2
ILMN_9320	AKAP12	rs6909279	C,G	0,60	-0,08	0,06	1,5E-01	bmp2
ILMN_941	C6orf211	rs6909279	C,G	0,60	-0,03	0,02	1,5E-01	bmp2
ILMN_9320	AKAP12	rs6909279	C,G	0,60	-0,06	0,04	1,7E-01	dex
ILMN_23727	MAL2	rs7839059	A,C	0,32	0,02	0,01	1,7E-01	bmp2
ILMN_23727	MAL2	rs7839059	A,C	0,32	0,01	0,01	1,9E-01	pge2
ILMN_18218	C6orf97	rs6909279	C,G	0,60	0,02	0,01	2,0E-01	pge2
ILMN_18367	FMN2	rs9287237	G,T	0,84	0,16	0,12	2,0E-01	untreated
ILMN_27754	EPSTI1	rs1021188	C,T	0,15	0,17	0,14	2,1E-01	dex
ILMN_15564	ZBTB2	rs6909279	C,G	0,60	0,02	0,02	2,1E-01	dex
ILMN_941	C6orf211	rs6909279	C,G	0,60	-0,03	0,03	2,2E-01	dex
ILMN_12003	AKAP11	rs1021188	C,T	0,15	-0,03	0,03	2,3E-01	dex
ILMN_12003	AKAP11	rs1021188	C,T	0,15	-0,03	0,02	2,4E-01	bmp2
ILMN_24099	EYA4	rs271170	C,T	0,70	-0,08	0,07	2,5E-01	pge2
ILMN_13487	RPS12	rs271170	C,T	0,70	0,02	0,02	2,8E-01	pge2
ILMN_15564	ZBTB2	rs6909279	C,G	0,60	0,02	0,02	3,2E-01	untreated
ILMN_24616	GREM2	rs9287237	T,G	0,84	-0,08	0,08	3,2E-01	bmp2
ILMN_14698	FLJ40919	rs1021188	C,T	0,15	-0,01	0,01	3,3E-01	pge2
ILMN_18367	FMN2	rs9287237	G,T	0,84	0,12	0,13	3,4E-01	bmp2
ILMN_29019	C6orf96	rs6909279	C,G	0,60	-0,02	0,02	4,1E-01	untreated
ILMN_16767	DGKH	rs1021188	C,T	0,15	0,00	0,01	4,4E-01	untreated
ILMN_18218	C6orf97	rs6909279	C,G	0,60	0,01	0,02	4,6E-01	untreated
ILMN_13487	RPS12	rs271170	C,T	0,70	0,01	0,02	4,8E-01	untreated
ILMN_21404	NOV	rs7839059	A,C	0,32	-0,07	0,10	4,9E-01	pge2
ILMN_1354	TNFSF11	rs1021188	C,T	0,15	0,00	0,00	5,0E-01	bmp2
ILMN_20988	MTHFD1L	rs6909279	C,G	0,60	0,04	0,06	5,0E-01	pge2
ILMN_6397	COLEC10	rs7839059	A,C	0,32	0,00	0,00	5,1E-01	bmp2
ILMN_29019	C6orf96	rs6909279	C,G	0,60	0,01	0,02	5,3E-01	dex
ILMN_24616	GREM2	rs9287237	T,G	0,84	-0,04	0,06	5,3E-01	untreated
ILMN_16767	DGKH	rs1021188	C,T	0,15	0,00	0,01	5,7E-01	dex
ILMN_12003	AKAP11	rs1021188	C,T	0,15	0,01	0,02	5,8E-01	pge2
ILMN_27754	EPSTI1	rs1021188	C,T	0,15	-0,07	0,13	5,9E-01	pge2
ILMN_15142	ESR1	rs6909279	C,G	0,60	-0,01	0,01	6,2E-01	bmp2
ILMN_27754	EPSTI1	rs1021188	C,T	0,15	0,08	0,16	6,2E-01	untreated
ILMN_15142	ESR1	rs6909279	C,G	0,60	-0,01	0,01	6,3E-01	dex
ILMN_16565	VNN2	rs271170	C,T	0,70	-0,01	0,03	6,4E-01	bmp2
ILMN_21404	NOV	rs7839059	A,C	0,32	0,05	0,11	6,4E-01	untreated
ILMN_14698	FLJ40919	rs1021188	C,T	0,15	0,00	0,01	6,5E-01	dex
ILMN_29019	C6orf96	rs6909279	C,G	0,60	-0,01	0,02	6,5E-01	pge2
ILMN_16565	VNN2	rs271170	C,T	0,70	-0,01	0,03	6,6E-01	untreated
ILMN_20988	MTHFD1L	rs6909279	C,G	0,60	0,02	0,05	6,7E-01	dex
ILMN_6495	TNFRSF11B	rs7839059	A,C	0,32	0,07	0,18	7,0E-01	pge2
ILMN_6495	TNFRSF11B	rs7839059	A,C	0,32	0,06	0,15	7,1E-01	bmp2
ILMN_941	C6orf211	rs6909279	C,G	0,60	-0,01	0,02	7,2E-01	pge2
ILMN_6495	TNFRSF11B	rs7839059	A,C	0,32	0,06	0,17	7,2E-01	untreated
ILMN_15564	ZBTB2	rs6909279	C,G	0,60	0,00	0,01	7,3E-01	pge2
ILMN_6495	TNFRSF11B	rs7839059	A,C	0,32	0,05	0,16	7,3E-01	dex
ILMN_20988	MTHFD1L	rs6909279	C,G	0,60	0,02	0,05	7,8E-01	untreated
ILMN_6397	COLEC10	rs7839059	A,C	0,32	0,00	0,00	7,8E-01	untreated
ILMN_21404	NOV	rs7839059	A,C	0,32	-0,03	0,11	7,9E-01	bmp2
ILMN_1354	TNFSF11	rs1021188	C,T	0,15	0,00	0,00	7,9E-01	pge2
ILMN_15142	ESR1	rs6909279	C,G	0,60	0,00	0,01	8,1E-01	pge2
ILMN_15142	ESR1	rs6909279	C,G	0,60	0,00	0,01	8,2E-01	untreated
ILMN_16565	VNN2	rs271170	C,T	0,70	-0,01	0,04	8,2E-01	dex
ILMN_16565	VNN2	rs271170	C,T	0,70	-0,01	0,05	8,5E-01	pge2
ILMN_15564	ZBTB2	rs6909279	C,G	0,60	0,01	0,03	8,5E-01	bmp2
ILMN_29019	C6orf96	rs6909279	C,G	0,60	0,00	0,02	8,5E-01	bmp2
ILMN_13487	RPS12	rs271170	C,T	0,70	0,00	0,02	8,6E-01	bmp2
ILMN_13487	RPS12	rs271170	C,T	0,70	0,00	0,02	8,6E-01	dex
ILMN_12003	AKAP11	rs1021188	C,T	0,15	0,00	0,02	8,9E-01	untreated
ILMN_20988	MTHFD1L	rs6909279	C,G	0,60	0,01	0,04	9,0E-01	bmp2
ILMN_1354	TNFSF11	rs1021188	C,T	0,15	0,00	0,00	9,1E-01	untreated
ILMN_27754	EPSTI1	rs1021188	C,T	0,15	-0,01	0,15	9,4E-01	bmp2
ILMN_1354	TNFSF11	rs1021188	C,T	0,15	0,00	0,01	9,6E-01	dex
ILMN_16767	DGKH	rs1021188	C,T	0,15	0,00	0,01	9,8E-01	bmp2
ILMN_6397	COLEC10	rs7839059	A,C	0,32	0,00	0,00	9,9E-01	dex

SNPs associated with vBMD at the genome-wide significance level as reported here were tested for association with resting or induced gene expression of neighbouring gene transcripts, in primary human osteoblasts derived from 113 (51 female and 62 male donors, respectively) unrelated Swedish donors.
