# Supplementary material for: Genetic Determinants of Trabecular and Cortical Volumetric Bone Mineral Densities and Bone Microstructure
Source: PLoS Genet. 2013 Feb 21;9(2):e1003247. doi: 10.1371/journal.pgen.1003247 (PMC3578773; doi:10.1371/journal.pgen.1003247)
Supplement: Table S6 — Characteristics of the MrOS Sweden fracture cohort. (PDF) [file pgen.1003247.s006.pdf]

Table S6 Characteristics of the MrOS Sweden fracture cohort

	**MrOS Sweden Fracture cohort** (n=3014)	
	mean	sd
Age, years	75,4	3,2
Men, %	100	
Height, cm	174,8	6,5
Weight, kg	80,8	12,1
Subjects with validated incident fractures	*(n=3014)*	
All fractures	388	(23,7)
Hip	90	(5,2)
Prevalent Vertebral Fractures Among Men with an X-ray at baseline	*(n=1445)*	
≥ 1 Fracture	225	(15,6%)

Values are given as mean ± SD. For incident fractures, the numbers of subjects with first fractures are given with the incidence/1000 person-years shown within parentheses. For prevalent X-ray verified vertebral fractures, the number of subjects with at least one fracture is given with percentage of the subjects within parentheses.
