# Supplementary material for: Genetic Determinants of Trabecular and Cortical Volumetric Bone Mineral Densities and Bone Microstructure
Source: PLoS Genet. 2013 Feb 21;9(2):e1003247. doi: 10.1371/journal.pgen.1003247 (PMC3578773; doi:10.1371/journal.pgen.1003247)
Supplement: Table S7 — Associations between cortical and trabecular vBMD SNPs and fractures in MrOS Sweden. (PDF) [file pgen.1003247.s007.pdf]

Table S7 Associations between cortical and trabecular vBMD SNPs and fractures in MrOS Sweden

	**Cortical vBMD SNPs**				**Trabecular vBMD SNP**
	rs1021188	rs271170	rs7839059	rs6909279	rs9287237
Effect allele	C	T	A	G	T
Incident fractures	*n=2885*	*n=2821*	*n=2820*	*n=2831*	*n=2827*
All fractures (HR per effect allele)	0,82 (0,66-1,02)	0,98 (0,83-1,15)	1,10 (0,94-1,28)	1,03 (0,88-1,19)	0,75 (0,60-0,93)
Hip (HR per effect allele)	0,69 (0,43-1,11)	1,10 (0,79-1,53)	1,01 (0,73-1,41)	1,31 (0,96-1,78)	0,59 (0,36-0,98)
Prevalent Vertebral Fractures	*n=1375*	*n=1333*	*n=1329*	*n=1334*	*n=1333*
≥ 1 Fracture (OR per effect allele)	0,85 (0,63-1,15)	0,97 (0,77-1,23)	1,12 (0,89-1,40)	1,12 (0,91-1,39)	0,68 (0,50-0,94)

Hazard ratios (HR) and odds ratio (OR) per effect allele are given with 95% confidence intervals within parentheses. The models are adjusted for age, height, weight and center.
